# Prediction of sonochemical activity based on dimensionless analysis and multivariate linear regression

**DOI:** 10.1016/j.ultsonch.2025.107427

**Published:** 2025-06-12

**Authors:** Yucheng Zhu, Xueliang Zhu, Irem Soyler, Xuhai Pan, Lian X. Liu, Madeleine J. Bussemaker

**Affiliations:** a School of Chemistry and Chemical Engineering, University of Surrey, Guildford, United Kingdom; b College of Safety Science and Engineering, Nanjing Tech University, Nanjing, China

**Keywords:** Sonochemical activity, Multi-parameter coupling, Dimensionless numbers, Mathematical modelling framework

## Abstract

Acoustic cavitation is influenced by multiple variables, including ultrasound frequency, power, and reactor configuration, all of which exert interactive and nonlinear effects on the performance of sonochemical systems. Current research on parametric effects has predominantly focused on the general trends of individual parameters, with limited investigation into their combined and interdependent influences. In this study, the sonochemical activity was systematically measured using sonochemiluminescence (SCL) and potassium iodide (KI) dosimetry under over 110 distinct operating conditions. The experimental data were transformed into dimensionless form to examine multi-parameter coupling effects in ultrasonic processes. Through theoretical analysis and experimental validation, seven dimensionless numbers (*Π1–Π7*) were identified, elucidating the roles of bubble dynamics, cavitation environment, acoustic wave propagation, and thermal effects in sonochemical processes. A dimensionless multivariate regression model framework was then developed to predict sonochemical activity under varying operating conditions. The applicability and generalizability of the model were verified by comparing predictive results with published studies. The study is the first to systematically integrate dimensionless analysis with multivariate regression, enabling a comprehensive exploration of multi-parameter coupling effects on sonochemical activity and establishing a predictive mathematical modelling framework. This deepens the understanding of the dynamics of ultrasonic cavitation and lays the foundation for future applications in more complex fluid systems, non-aqueous media, and different types of ultrasound equipment. Therefore, this study represents an initial step toward advancing multi-parameter optimisation strategies in sonochemistry, offering a novel framework for systematically optimising sonochemical systems and guiding future experimental and industrial applications.

## Highlights

• Seven dimensionless numbers were proposed to describe sonochemical systems.
• A predictive framework was built using the defined dimensionless groups.
• The framework reveals multiparametric interactions in sonochemical processes.
• Dimensionless groups enhance the transferability of the model to various systems.
• Dimensionless modelling strategy was advanced toward sonochemical applications.

## Nomenclature

*A_SA_*: total pixels’ number in the selected area, [-]
*A_TR_*: the number of pixels for the total reactor cross-section, [-]
*A*_t_: transducer plate-liquid contact area, [m^2^]
*c_i_*: the saturation concentration of *i* at the bubble wall, [mol m^−3^]
*c_0,i_*: the concentration of *i* at *r*=∞ [mol m^−3^]
*c*_L_: speed of sound, [m s^−1^]
*Cp*: specific heat capacity, [J kg^−1^·K^−1^]
*D*: diffusion coefficient, [ m^2^ s^−1^]
*f*: frequency, [kHz]
*GS_SA_*: sum of the greyscales of all pixels in the selected area, [-]
*H_R_*: liquid height, [m]
*I_A_*: acoustic intensity, [W m^−2^]
*I_diff_*: the diffusion length, [m]
*k_φ_*: Arrhenius-coefficient of reaction *φ* [m^3^ mol^−1^·s^−1^]
*m*: mass, [kg]
${\dot{m}}_{\mathrm{eva}}$: evaporation rate, [kg s^−1^]
${\dot{m}}_{\mathrm{con}}$: condensation rate, [kg s^−1^]
*n_i_*: number of i molecules inside the bubble
*n_w_*: number of vapour molecules inside the bubble, [-]
*n_t_*: total number of molecules inside the bubble, [-]
*N_i_*: the rate of change in the number of molecules of the component, [mol s^−1^]
*N_A_*: Avogadro constant, [l·mol^−1^]
${\dot{N}}_{w}$: the change rate of molecule number of vapour
p_v_*: saturated pressure of vapour at *T_0_*, [Pa]
*p_i_*: partial pressure of component *i* within the bubble, [Pa]
*P_A_*: sound pressure amplitude, [Pa]
*P_B_*: bubble internal pressure, [Pa]
*P_0_*: initial static pressure
*P_∞_*: static ambient pressure, [Pa]
*P_∞_(t)*: far-field pressure, [Pa]
*P_eff_*: effective SCL proportion, [%]
*P_E_*: pressure inside the bubble at equilibrium, [Pa]
*P_L_*: liquid pressure, [Pa]
*P_V_*: vapour pressure inside the bubble, [Pa]
*r_φ_*: rate of reaction *φ,* [mol m^−3^·s^−1^]
*r_i,prod_*: the cumulative reaction rates in which *i* is involved as a product, [mol m^−3^·s^−1^]
*r_i,destr_*: the cumulative reaction rates in which *i* is involved as a reactant, [mol m^−3^·s^−1^]
*R*: bubble radius, [m]
*R_E_*: equilibrium radius of the bubble, [m]
*R_v_*: specific gas constant of water vapour, [J kg^−1^·K^−1^]
*R_r_*: resonance radius for bubble, [m]
$\dot{R}$: first time derivative of *R*, [m s^−1^]
$\ddot{R}$: second time derivative of *R*, [m s^−2^]
*R*_g_: gas constant, [J mol^−1^·K^−1^]
*t*: time, [s]
*T*: temperature, [K]
*V*: volume, [m^3^]

**Greek symbols**
*γ*: adiabatic factor, [-]
*μ_L_*: dynamic viscosity of the liquid, [Pa·s]
*σ*: surface tension, [N m^−1^]
*Φ*: overall SCL intensity, [-]
*α_M_*: accommodation coefficient for evaporation, [-]
*Γ*: correction factor, [-]

**Subscripts**
*∞*: ambient
0: initial state
*φ*: index for chemical reaction
i: component
L: liquid phase
t: total
w: water
SA: selected area
TR: total reactor
eff: effective SCL

## 1 Introduction

Due to cavitation, sonochemical processes offer a distinctive ability to conduct high-energy chemistry in aqueous systems without chemical additives or catalysts [1]. Sonochemical processing is increasingly recognized as a sustainable alternative or a process intensification approach across various applications, including removal of organic pollutants [2], the processing of biomass [3,4], the advancement of emerging materials [5], and the destruction of pharmaceutical waste [6].

The basic mechanism of the sonochemical process is ultrasonically induced transient cavitation: micro-nuclei in the liquid violently collapse due to sound pressure, resulting in the immediate formation of extremely high temperatures and pressures, along with the production of reactive oxidant species [7]. The active chemicals are released into the solution by diffusion through the bubble surface or bubble collapse, which promotes chemical reactions [8]. Iodide dosimetry and sonochemiluminescence (SCL) measurements are commonly used to assess the sonochemical activity of acoustic reactors [[9], [10], [11]]. The distinction in sonochemical reactivity obtained via these two measurements is speculated to arise from the different properties of the target solutes [8]. Both forms of sonochemical activity are susceptible to variations in reaction parameters [7,12]. Therefore, unravelling the parametric effects in sonochemistry, including SCL emission and dosimetry activities, is crucial for advancing effective sonochemical processes and devices.

The performance of an acoustic system is influenced by various interdependent and nonlinear factors, including operational parameters such as ultrasonic conditions (e.g., acoustic frequency, sound pressure amplitude) [7], medium characteristics (e.g., viscosity and density of the solution) [13], and reactor geometry (e.g., shape and diameter of the reactor) [4]. These factors collectively affect bubble dynamics and chemical reaction kinetics within the reaction system, thereby further affecting the subsequent sonochemical activity [14]. Several studies have reviewed the parametric effects on sonochemical activity in aqueous systems [7,[15], [16], [17]].

Nonlinear oscillations at high amplitudes facilitate bubble fragmentation, leading to more active bubbles. Increases in pressure amplitude also increase the cavitation temperature, and can boost the chemical yield [18,19]. Once the pressure amplitude surpasses the maximum limit of the cavitation threshold, it leads to bubble coalescence and degassing, thus reducing reaction efficiency [20]. Bubbles display larger sizes and more violent collapses at low frequencies, yet lower frequencies are associated with fewer bubbles. Reactor solutions exposed to this frequency range, i.e. *f*＜200 kHz, are generally prone to provide physical effects [21]. High frequencies, i.e. *f* ≥ 200 kHz, enhance reaction efficiency and are conducive to improved overall chemical yield [7,19]. However, at ultrahigh frequencies exceeding 1 MHz, the smaller bubble size and higher cavitation threshold make it more difficult for bubbles to reach the cavitation threshold, reducing the number of active bubbles and ultimately inhibiting cavitation [22].

Ultrasonic parameters are consistently investigated as the primary parameters (e.g., pressure amplitude and frequency), while certain configurations or environmental parameters are investigated as secondary parameters (e.g., solid addition and liquid properties). It is reported that modifying liquid height might optimise bubble distribution, while reflecting plates could enhance standing wave components, resulting in enhancing local reaction efficiency [[23], [24], [25]]. Utilising liquids with higher surface tension necessitates more pressure for the nucleation of cavitation bubbles [26,27]. However, the high surface tension exerts a reduced restrictive barrier to bubble growth and enhances stability during bubble expansion [28]. Additional liquid properties also affect cavitation intensity. Typically, high-viscosity liquids are considered to reduce bubble oscillation frequency and thus alleviate cavitation activity [29]. However, under specific conditions, increased viscosity may also enhance the intensity of bubble collapse and acoustic emissions [30]. This suggests that the role of viscosity in cavitation dynamics is context-dependent and may vary with acoustic pressure and frequency. In addition, a solution with higher vapour pressure will diminish the energy released during collapse (cushioning effect) and hamper cavitation intensity [31].

The optimisation of sonochemical reactor parameters primarily relies on a qualitative evaluation of individual parameter effects rather than a holistic overview of a combined set of parameters [21]. While these studies provide valuable insights into key factors, their practical applicability is limited due to the complex synergistic and antagonistic interactions among multiple parameters in real-world systems [32,33]. Conventional univariate approaches fail to capture these interactions, whereas full factorial studies demand significant experimental resources [33]. Despite these challenges, advancing multi-parameter optimisation is crucial for transitioning ultrasound-based technology from laboratory-scale refinement to practical industrial applications [34].

Dimensionless analysis is an attractive approach for studying multi-parameter systems and has been widely applied in fields such as fluid mechanics [35] and heat transfer [36,37]. However, its systematic application in sonochemistry remains limited. Early models, such as the acoustic cavitation number [38] and its modified versions [33], demonstrated the feasibility of using multiple physical parameters to describe and predict cavitation. Nonetheless, these models remain relatively simplistic and often fail to capture the spatial distribution of the acoustic field, the variability of fluid properties with temperature and pressure, and potential chemical effects occurring in ultrasonic systems. As a result, their applicability under complex or dynamic conditions is constrained.

The present work thus attempts to elucidate the fundamental multi-parameter effects in sonochemistry by proposing a novel approach that combines dimensionless analysis with multivariate regression. An acoustic reaction system, equipped with an interchangeable plate transducer [39], was used to investigate sonochemical activity under varying frequencies, solution volumes, and pressure amplitudes. Quantitative analysis of sonochemical reactions was conducted, and the fundamental physical factors associated with ultrasound were identified based on bubble dynamics and chemical reaction kinetics. Key parameters, (e.g. ultrasonic frequency (*f*), speed of sound (*c*), and solution viscosity (*σ*), etc.), were selected and transformed into dimensionless groups using the Buckingham Pi theorem [40]. This transformation eliminates dimensional dependence and reveals potential scaling laws. The multiple parametric framework was extended by systematically examining key physical parameters in the sonochemical system. This extension incorporates acoustic field characteristics, fluid properties that influence bubble dynamics, and chemical reaction kinetics. A set of dimensionless parameters was developed and—for the first time—combined with multiple linear regression to construct a predictive model for sonochemical activity. This model enables the estimation of targeted sonochemical activities, i.e. ultrasonic KI dosimetry activity and SCL activity under given initial system conditions, providing theoretical guidance for pre-selecting optimal parameter combinations. Validation through literature case studies [[8], [9], [10],^41^] demonstrates the model’s general applicability and robust predictive performance. This approach introduces a new perspective for the optimized design of sonochemical reactions, paving the way for more efficient parameter selection and advanced sonochemistry.

## 2 Experiment and analytical methodology

### 2.1 Ultrasonic set-up

**Fig. 1:**
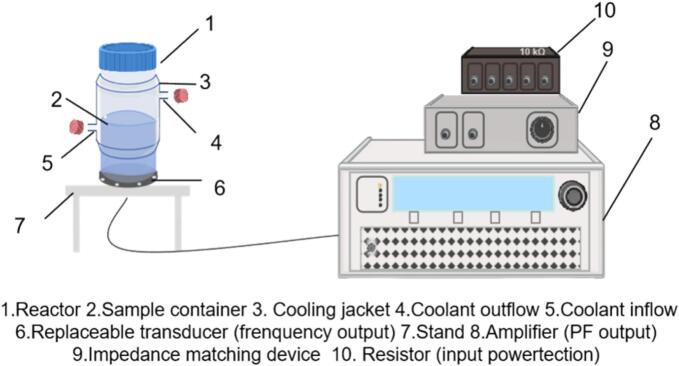
Schematic diagram of the ultrasonic system.

A single-signal sonication system with a plate transducer (Fig. 1) was used in our work, and the configuration details have been described in previous work [39]**.** According to the electric power response, we ultimately performed the test across 117 and 114 operating conditions with KI dosimetry and SCL, respectively, as detailed in Table 1.

**Table 1:** Experimental details (test sets).

Transducer number	Frequency (kHz)	Loading power (W)^a^	Liquid height (mm)^b^
Multi-frequency 1	22	10, 20, 30, 40	56.7, 85.1, 113.5
	44	10, 20, 30, 40	56.7, 85.1, 113.5
	98	10, 20, 30, 40	56.7, 85.1, 113.5
	128	10, 20, 30, 40	56.7, 85.1, 113.5
Single-frequency 2	200	10, 20, 30, 40	56.7, 85.1, 113.5
Single-frequency 3	400	10, 20, 30, 40	56.7, 85.1, 113.5
Single-frequency 4	500	10, 20, 30, 40	56.7, 85.1, 113.5
Single-frequency 5	760	10, 20, 30, 40	56.7, 85.1, 113.5
Single-frequency 6	1000	10, 20, 30^c^	56.7, 85.1, 113.5
Single-frequency 7	2000	10, 20, 30, 40	56.7, 85.1, 113.5

a Loading power refers to the electrical input to the ultrasonic transducer.

b The liquid heights of 56.7, 85.1, and 113.5 mm correspond to reactor volumes of 200, 300, and 400 mL, respectively, based on a reactor inner diameter of 67 mm.

c Due to the response of the device, a frequency of 1000 kHz and a load power of 40 W were not tested.

Throughout this work, loading power refers to the electrical input to the ultrasonic transducer. This differs from calorimetric power, which represents the acoustic energy absorbed by the liquid [42]. Loading power was used for experimental control and data analysis.

### 2.2 Potassium iodide (KI) dosimetry

Ultrasound irradiation of an aqueous KI solution results in the oxidation of particular iodide (I^−^) to diiodide (I_2_) by free radicals generated from the collapse of cavitation bubbles. The left-over iodine then combines with I_2_ to generate triiodide (I_3_^−^) as the end product (Eq. (1)).

(1) $$I_{2}+I^{-}\to I_{3}^{-}$$

The other typical reaction during ultrasonic irradiation seems to adhere to the way given as follows:

(2) $$H_{2}O\left(\left(\left(\;\right)\right)\right)\mapsto H\cdot +OH\cdot ,$$

(3) $$\mathrm{HO}\cdot +I^{-}\to OH^{-}+I\cdot ,$$

(4) $$I\cdot +I^{-}\to I_{2}^{-},$$

(5) $$H_{2}O_{2}+2I^{-}\to 2OH^{-}+I_{2}.$$

Where the symbol ‘)))’ indicates a reaction a response triggered by ultrasonic cavitation.

The reaction (5) requires catalytic conditions for its execution; hence, the solutions with and without the catalyst (0.5 mM (NH_4_)_6_Mo_7_O_24_ in this study) can be used to investigate the two different oxidation capabilities of the reactor induced by ultrasound. The outcomes derived from ultrasonic irradiation of a 0.1 M KI solution could indicate the generation of iodide oxidation radicals (IORS). Ultrasound operating on a KI solution with a particular catalyst allows for IORS and H_2_O_2_. The I_3_^−^ production could be computed from the absorbance by a UV–Vis (Thermo Scientific Evolution 201 UV–visible spectrometer) at 350 nm. This work attempts to improve the generalisability of the research findings through dimensionless analysis. Consequently, in assessing the dosimetry results, the dimensionless results, i.e. absorbance values, are used directly to reflect the oxidative properties of ultrasound without additional computation. It is important to note that the generation of free radicals through sonication follows a zero-order reaction kinetic model [10]. In this work, the absorbance of the solution was measured after 10 min of ultrasonic irradiation.

### 2.3 Sonochemiluminescence (SCL) intensity measurement

**Fig. 2:**
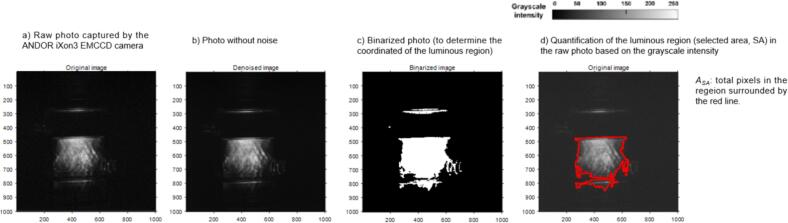
Diagram of image processing [39].

The spatial distribution and intensities of SCL emission during ultrasonic irradiation were recorded at ambient temperature in a 0.1 M NaOH solution containing 1 mM luminol. A minimum of three images per setting were captured and average results considered**.** The digital data for the images used in the analysis were obtained using an area-selective method (Fig. 2) to broaden the range of usable parameters [39]. Unlike previous methods that directly analyzed the grayscale values of luminescent regions, this study first applies a normalization process to the image grayscale data to get some dimensionless results and ensure the consistency and comparability of data under different imaging conditions. The following section introduces the normalization method and its mathematical expression.

The overall SCL intensity (*Φ*) is described as the total greyscale level within the selected area (active area within the red line, Fig. 2) and is computed through Eq. (6).

(6) $$\Phi =\frac{GS_{\mathrm{SA}}}{2^{b}}$$

Where, *GS_SA_* is the sum of the greyscales of all pixels in the selected area, *b* represents the bit depth of the image, which is the number of bits per pixel, and *2^b^* reflects the maximum grayscale value for the given bit depth.

The effective SCL proportion (*P_eff_*) is defined as the active SCL region as a fraction of the total reactor cross-section and is calculated via Eq. (7).

(7) $$P_{\mathrm{eff}}=\frac{A_{\mathrm{SA}}}{A_{\mathrm{TR}}}\times 100\%$$

Where*, A_SA_* is total pixels’ number in the active SCL region selected and *A_TR_* is the number of pixels for the total reactor cross-section.

It is important to note that no global brightness normalization was applied to the image set, as grayscale data were extracted directly from the original images captured by the camera. This deliberate choice preserves the physical differences in SCL signal intensity across different operational conditions. As a result, variations in the brightness of background regions (i.e., areas without actual SCL emission) may appear across images. This approach ensures that the extracted data reflect actual emission conditions rather than artifacts introduced during image processing.

### 2.4 Modelling and validation using multiple linear regression (MLR)

The physical-driven models were developed based on dimensionless analysis and multiple linear regression (MLR). A set of dimensionless numbers governing cavitation and sonochemical activity was identified by analysing the key physical quantities related to bubble dynamics and reaction kinetics in an acoustic system combined with Buckingham's *Pi* theorem [40]. The values of these dimensionless numbers were computed using MATLAB. To establish predictive models for sonochemical activity, empirical correlations were formulated through MLR, using statistical fitting functions available in Origin. The coefficient of determination, R^2^, was used to assess the predictive accuracy and explanatory power of the physical-driven models.

The model's generalizability was studied through comparison to previously published studies [[8], [9], [10],^41^]. Proposed models were used to forecast the sonochemical activities using initial operating parameters in these published works, then the comparisons were made to published data. To further validate the model’s applicability across different reactor configurations, a cubic reactor was used for additional SCL measurements. The reactor featured a side length of 11 cm and was made of plastic, distinguishing it from the original setup. The transducer and vibration plate used in the cubic reactor were of the same size and material as those in the modelling study. Notably, in this configuration, the vibration plate was positioned at the centre of the reactor's bottom rather than covering the entire base. The experimental configurations used to test the model are shown in the Appendix A (Table S1), and a schematic diagram of the cubic configuration and the experimental results are shown in Appendix B (Figs. S1 and S2).

## 3 Theoretical consideration

Sonochemistry exhibits unique physicochemical effects, driven by acoustic cavitation resulting from ultrasound propagation in liquids. The core of the sonochemical mechanism lies in the dynamic behaviour of cavitation bubbles and the free radical chemical reactions generated during cavitation [43]. The oscillation and collapse of cavitation bubbles determine the extreme local conditions, while reaction kinetics govern the formation, diffusion, and interaction of radicals with solutes.

### 3.1 Mechanism of sonication

#### 3.1.1 Bubble dynamics

The formation of extreme local conditions can be understood through bubble dynamics, examining the nucleation, growth, and collapse of transient cavitation bubbles. The Keller-Miksis equation (Eq. (8)) describes the radial dynamics of bubbles in liquid medium [14,16,44]. The equation expands upon the Rayleigh-Plesset formulation by incorporating supplementary variables to account for compressibility effects in liquids and bubbles.

(8) $$\left(1-\frac{\dot{R}}{c_{L}}\right)R\ddot{R}+\frac{3}{2}\left(1-\frac{\dot{R}}{3c{\;}_{L}}\right){\dot{R}}^{2}=\frac{1}{\rho_{L}}\left(1+\frac{\dot{R}}{c_{L}}+\frac{R}{c_{L}}\frac{d}{\mathrm{dt}}\right)\left[P_{L}-P_{\infty }\left(t\right)\right].$$

*R* denotes the instantaneous bubble radius, *c_L_* is the sound speed in the liquid, and *ρ_L_* is the density of the liquid. $\dot{R}$ and $\ddot{R}$ are the first and second time derivatives of the bubble radius, respectively, indicating the velocity of expansion and shrinking, and the acceleration of the bubble wall. Additionally, the far-field pressure *P_∞_(t)* has static and dynamic components expressed as

(9) $$P_{\infty }\left(t\right)=P_{\infty }+P_{A}\sin\left(2\pi ft\right).$$

Where *P_∞_* is the static ambient pressure, *P_A_* and *f* are the sound pressure amplitude and frequency of the exciting irradiation, respectively. *P_A_* is correlated with the acoustic intensity *I_A_*, as power per unit area, as [45]

(10) $$\frac{P_{A}^{2}}{2}=I_{A}\rho_{L}c_{L}.$$

The liquid pressure (*P_L_*) at the bubble wall is correlated with the internal pressure (*P_B_*) as

(11) $$P_{L}=P_{B}-\frac{2\sigma }{R}-4\mu_{L}\frac{\dot{R}}{R}.$$

Where *σ* is the surface tension and *μ_L_* is the dynamic viscosity of the liquid. The gas inside the bubble is considered an ideal gas, with pressure (*P_B_*) correlated to total number of molecules inside the bubble (*n_t_*), temperature (*T*), and volume (*V*) via the state equation as

(12) $$P_{B}V=n_{t}R_{g}T.$$

Many studies assume that the expansion phase of the bubble is isothermal [16,46], and the collapse phase is deemed adiabatic due to the exceedingly brief bubble oscillation duration in the ultrasonic field [7,47,48]. In addition, the bubble temperature is assumed to be spatially uniform although the actual highest point mostly at the bubble core [46]. Since the thermal transfer in the boundary layer has little impact on the main research objectives of ultrasonic reactivity, it was neglected. According to the aforementioned assumptions, the temperature (*T*) and pressure during the bubble expansion phase can be articulated as [49]

(13) $$T=T_{\infty }=T_{0},$$

(14) $$P_{B}=P_{V}+\left(P_{\infty }+\frac{2\sigma }{R_{0}}-P_{V}\right){\left(\frac{R{\;}_{0}}{R}\right)}^{3}.$$

Where, *T_0_* is the initial temperature of the liquid which is equal to the ambient temperature (*T_∞_*). And *P_V_* is vapour pressure inside the bubble. Based on the adiabatic mechanism, the equations for temperature and pressure become:

(15) $$T=T_{\infty }{\left(\frac{R_{\max}}{R}\right)}^{3\left(\gamma -1\right)},$$

(16) $$P_{B}=\left[P_{V}+\left(P_{0}+\frac{2\sigma }{R_{0}}\right){\left(\frac{R_{0}}{R_{\max}}\right)}^{3}\right]{\left(\frac{R_{\max}}{R}\right)}^{3\gamma }.$$

Where *R_max_* is the maximum radius the bubble reaches during expansion, and γ is the adiabatic factor, typically approximated as 1.4 based on the gases contained within the bubble [50].

#### 3.1.2 Chemical reaction kinetics

Studying reaction kinetics enables understanding of radical diffusion behaviour and the chemical interactions across different regions of the reactor, including the bubble interior, the gas–liquid interface, and the liquid phase [16]. The chemical reaction *φ* taking place inside the bubble can be simply expressed as

(17) φ:A+B→C+D.

Herein, *A* and *B* are designated as reactants, while *C* and *D* are referred to as products. The reactions that take place always follow the law of conservation of mass. The reaction *φ*’s rate, related to unit time and volume, can be calculated by applying the modified Arrhenius equation as

(18) $$r_{\varphi }=k_{\varphi }\left[A\right]\left[B\right]=A_{\varphi }T^{b_{\varphi }}e^{-\frac{c_{\varphi }}{T}}\left[A\right]\left[B\right].$$

Where, *r_φ_* is the rate of reaction *φ*, *k_φ_* is the Arrhenius-coefficient of reaction *φ*, and [*i*] is the concentration of component *i*. The quantities *A_φ_, b_φ_* and *c_φ_* are constants specific to reaction *φ*, representing the pre-exponential factor, the temperature exponent, and the activation-related constant, respectively. In the following, the subscript *i* denotes the variable of component *i*. When a component is involved in multiple chemical reactions, the change in its molecular quantity should be evaluated by considering its roles as a reactant, a product, and the effects of diffusion. Consequently, the rate of change in the number of molecules of the component (*N_i_*) can be stated as

(19) $${\dot{N}}_{i}=V\sum \left(r_{i,prod}-r_{i,destr}\right)+A{\left(\frac{\mathrm{dm}}{\mathrm{dt}}\right)}_{i},$$

Where *r_i,prod_* and *r_i,destr_* are the cumulative reaction rates in which *i* is involved as a product and a reactant, respectively; *A* and *V* are the surface area and volume of the bubble; respectively. And *(dm/dt)i* is the rate of diffusion of each component *i*. The rate of diffusion for each component *(dm/dt)i* is determined by [51]

(20) $${\left(\frac{\mathrm{dm}}{\mathrm{dt}}\right)}_{i}={\left(D{\left(\frac{\partial c}{\partial r}\right|}_{r=R}\right)}_{i}\approx D\frac{c_{0,i}-c_{i}}{l_{\mathrm{diff}}}.$$

Where *D* is the diffusion coefficient. *c_0,i_* is the concentration of *i* at *r*=∞, representing its concentration in the bulk liquid phase, far from the bubble. And *c_i_* is the saturation concentration of *i* at the bubble wall in liquid side defined by Henry’s law as

(21) $$c_{i}=\frac{10^{3}\rho_{L}N_{A}}{M_{w}K_{B}}p_{i}.$$

Where *N_A_* is the Avogadro-constant of substance, *K_B_* is the Henry-constant in water, *M_W_* is the molar mass of the solvent (e.g., water) and *p_i_* is the partial pressure of component *i* within the bubble, calculated through Dalton’s law as

(22) $$p_{i}=\frac{n_{i}}{n_{t}}P_{B},$$

Where *n_i_* is the number of *i* molecules inside the bubble. Furthermore, *l_diff_* in Eq.(20) is the diffusion length approximated via

(23) $$l_{\mathrm{diff}}=\min\left(\sqrt{\frac{\mathrm{RD}}{\left|\dot{R}\right|}},\frac{R}{\pi }\right).$$

During bubble oscillation, the water molecules within the bubbles undergo evaporation and condensation as a result of fluctuations in internal pressure and temperature. As the result, the change rate of molecule number of vapour could be expressed differently as

(24) $${\dot{N}}_{w}=V\sum \left(r_{w,prod}-r_{w,destr}\right)+A\dot{m},$$

Where $\dot{m}$ is the net evaporation rate for unit area and time described as

(25) $$\dot{m}={\dot{m}}_{\mathrm{eva}}-{\dot{m}}_{\mathrm{con}}.$$

Where ${\dot{m}}_{\mathrm{eva}}$ and ${\dot{m}}_{\mathrm{con}}$ denote the rate of evaporation and condensation, respectively and can be expressed as

(26) $${\dot{m}}_{\mathrm{eva}}=\frac{\alpha_{M}}{\sqrt{2\pi R_{v}}}\frac{p_{v}^{*}}{\sqrt{T_{0}}},$$

(27) $${\dot{m}}_{\mathrm{con}}=\frac{\alpha_{M}}{\sqrt{2\pi R_{v}}}\frac{\Gamma p_{v}}{\sqrt{T}},$$

Where *α_M_* is the accommodation coefficient for evaporation [52], *R_v_* is the specific gas constant of water vapour, p_v_* is the saturated pressure of vapour at *T_0_*, *Γ* is the correction factor and *p_v_* is the actual partial pressure of vapour inside the bubble, which can be described as

(28) $$p_{v}=\frac{n_{w}}{n_{t}}P_{B},$$

Where *n_w_* is the number of vapour molecules inside the bubble.

The beneficial effect of sonication on improving stoichiometry and catalysing chemical reactions arises from an intersection of cavitation, bubble dynamics, thermodynamics, and chemical processes [53,54].

### 3.2 Additional factors

Bubble behaviour in practical ultrasonic reactors is sensitive, complex, and non-linear to the acoustic field setting, including reactor configuration and control parameters [14]. Different responses affect sonochemical performance [7]. Here, additional potential parameter effects are briefly introduced and will be integrated with the mechanism-related parameters for further discussion.

#### 3.2.1 Specific heat capacity of liquid

During the retrospective phase of the sonication mechanism, heat transfer is not emphasised in the sonochemical process and is therefore neglected. Nonetheless, when an ultrasonic wave propagates through a liquid medium, a fraction of the energy supplied to the system is dissipated in the medium as heat [42]. This energy dissipation is directly affected by the Specific heat capacity of the liquid (*Cp*) of the solution. Several studies verify that the specific heat capacity of the liquid *Cp* significantly affects the sonochemistry and cavitation [13,42,55]. The influence of specific heat capacity *Cp* on acoustic pressure fields, cavitation active region volume, and acoustic streaming surface velocity has been systematically quantified through numerical studies involving twelve ionic liquids [13] Higher *Cp* were found to reduce the efficiency of acoustic energy concentration within the liquid medium, thereby decreasing cavitation zone size. This finding underscores the critical role of *Cp* in modulating acoustic wave propagation and cavitation energy distribution.

#### 3.2.2 Liquid height (reactor volume)

Liquid height (*H*_R_, or reactor volume) is recognized as a critical geometric parameter influencing sonochemical activity. Liquid height plays a crucial role in shaping the standing wave pattern of the acoustic field, which governs the spatial distribution and collapse dynamics of cavitation bubbles [23,25,56]. Sonochemical efficiency exhibits one or two distinct maxima at specific liquid heights within acoustic reactor [23]. Numerical simulations and experimental data further support this relationship, showing that standing waves and active cavitation fields emerge at these specific liquid levels [56].

Additionally, power intensity is inherently linked to the liquid volume through its definition, variation in liquid height affects the actual energy delivered per unit area. Although previous findings suggest that power density (W·L^−1^) and power intensity (W·cm^−2^) play a more decisive role than liquid height alone [41], incorporating liquid height into modelling remains essential for accurately capturing its influence on acoustic energy distribution and cavitation behaviour.

### 3.3 Buckingham’s *Pi* theorem and its application to sonochemistry

As reviewed above, many factors are associated with ultrasonic process, summarized in Table 2.

**Table 2:** Sonochemistry-related parameters.

Parameters	Abbrev.	SI unit	Base dimensional unit
Density of liquid	*ρ_L_*	kg·m^−3^	M·L^−3^
Number of total/ vapour molecules inside the bubble	*n_t/_ n_w_*	mol	N
Sound speed in the liquid	*c_L_*	m·s^−1^	L·T^−1^
Initial temperature of liquid	*T_0_*	K	Θ
Surface tension of liquid	*σ*	kg·s^−2^	M·T^−2^
Dynamic viscosity of liquid	*μ_L_*	kg·m^−1^·s^−1^	M·L^−1^·T^−1^
Static ambient pressure	*P_∞_*	kg·m^−1^·s^−2^	M·L^−1^·T^−2^
Acoustic intensity	*I_A_*	kg·s^−3^	M·T^−3^
Frequency of the exciting irradiation	*f*	s^−1^	T^−1^
Liquid height within the reactor	*H_R_*	m	L
Specific heat capacity of liquid	*C_p_*	m^2^·s^−2^·K^−1^	L^2^·T^−2^·Θ^−1^

The Buckingham *Pi* theorem [40] outlines a physical relationship *f(s_1_, s_2_,* …*, s_n_)* = 0, consisting of a collection of n physical quantities *s_1_*, *s_2_*, …, *s_n_*, each with distinct dimensions. Assuming that *j* dimensions of this set of physical quantities are independent and defined as the fundamental dimensions, this physical relationship can be expressed as a function of dimensionless variables, i.e., *f(Π1, Π2,* …*, Πk)* = 0. The number of dimensionless variables is represented by *k = n-j*. Since there are only 7 fundamental dimensions—length (L), mass (M), time (T), thermodynamic temperature (Θ), electric current (I), luminous intensity (J), and amount of substance (N)—the maximum possible value of j is 7.

The method of repeating variables is a streamlined approach to dimensional analysis [57]. With a set of essential recurring variables, the physical variables within the system are amalgamated into dimensionless numbers, thus simplifying the system's complexity [36]. This method involves selecting a subset of fundamental variables, referred to as repeating variables, based on their relevance to the system and their capacity to create independent dimensionless groupings. The repeating variables should collectively encompass all essential dimensions (M, L, T, etc.) inherent in the system and be mutually independent [57]. In this work, *H_R_, f, T_0_, n_t_, I_A_* are determined as repeating variables. Preliminary reviews indicate that they are key physical parameters that affect cavitation dynamics and sonochemical activity. Seven *Π* terms are thus determined in Table 3 based on Buckingham’s *Pi* theorem. The corresponding physical interpretations and classifications are also provided to assist with conceptual understanding. The validity and relevance of these dimensionless numbers are further evaluated and discussed in section 4.3.2 based on the modelling results.

**Table 3:** *Π* terms.

*Π* term	Expression	Physical meaning	Category
*Π1* = *ρ*_L_*H*_R_^a1^*f*^b1^*T*_0_^c1^*n*_w_^d1^*σ*^e1^	*ρ*_L_*H*_R_^3^*f*^2^/*σ*	Inertial-to-surface tension ratio	Bubble dynamics
*Π2* = *n_t_H*_R_^a2^*f*^b2^*T*_0_^c2^*n*_w_^d2^*σ*^e2^	*n*_w_/*n*_t_	Vapour content ratio in bubble	Cavitation environment
*Π3* = *cH*_R_^a3^*f*^b3^*T*_0_^c3^*n*_w_^d3^*σ*^e3^	*c*/*H*_R_*f*	Acoustic wavelength relative to reactor size	Acoustic wave transfer
*Π4* = *I*_A_*H*_R_^a4^*f*^b4^*T*_0_^c4^*n*_w_^d4^*σ*^e4^	*I*_A_/*fσ*	Acoustic intensity scaled by surface tension energy	Bubble dynamics
*Π5* = *μ*_L_*H*_R_^a5^*f*^b5^*T*_0_^c5^*n*_w_^d5^*σ*^e5^	*μ*_L_*H*_R_*f*/*σ*	Viscous damping contribution	Bubble dynamics
*Π6*=*P*_∞_*H*_R_^a6^*f*^b6^*T*_0_^c6^*n*_w_^d6^*σ*^e6^	*P*_∞_*H*_R_/*σ*	Ambient pressure influence	Cavitation environment
*Π7*=*C*_p_*H*_R_^a7^*f*^b7^*T*_0_^c7^*n*_w_^d7^*σ*^e7^	*C*_p_*T*_0_/(*H*_R_*f*)^2^	Thermal capacity and energy buffering	Thermal effect

All treatments were carried out in an open reactor at ambient temperature, with the highest temperature throughout the acoustic process remaining below 25 °C for all solutions (due to external cooling measures). Consequently, all parameters about the characteristics of the liquids during the fitting procedure were referenced to the property parameters of water at 293 K and 1 atm. Other fixed parameters involved and associated value are shown in Table 4.

**Table 4:** The parameter kept constant during the modelling.

Name	Abbrev.	Value	Unit
Density of liquid	*ρ_L_*	998.2	kg·m^3^
Specific heat capacity of liquid	*Cp*	4.186	kJ·kg^−1^·K^−1^
Sound speed in the liquid	*c*	1482.9	m/s
Dynamic viscosity of liquid	*μL*	0.001	Pa·s
Surface tension of liquid	*σ*	0.0728	N·m^−1^
Saturated vapour pressure	P_v_*	2338.8	Pa
Universal gas constant	*R_g_*	8.3146	J·mol^−1^·K^−1^
Avogadro-constant	*N_A_*	6.022 × 10^23^	1·mol^−1^
Transducer plate-liquid contact area	*A_t_*	35.26 × 10^−4^	m^2^

### 3.4 Estimation of molecular number (*Π2* calculations)

An active bubble is always assumed to be initiated from an equilibrium state, thus the corresponding equilibrium pressure is [16]

(29) $$P_{E}=P_{\infty }+\frac{2\sigma }{R_{E}}$$

Where *P_E_* is the pressure inside the bubble at equilibrium, and *R_E_* is the equilibrium radius of the bubble. When exposed to an external acoustic field, a bubble will respond with maximum amplitude when its oscillation frequency resonates with that of the acoustic field. Bubbles that attain the resonance size range expand to a maximum before collapsing, consequently leading to active cavitation, whereby the critical radius of the bubble can be considered as the resonance radius (*R_r_*) as [58,59]

(30) $$R_{\text{r}}=\frac{1}{2\pi f}\sqrt{\frac{3\gamma p_{\infty }}{\rho_{L}}}$$

Where *R_r_* is the resonance radius for bubble. For ease of understanding, we define the state before bubble collapse as quasi-equilibrium. We assumed only air (main active component is O_2_) and vapour molecules inside the bubble. The partial pressure of vapour inside a bubble when it reaches a critical size always be estimated by the saturated vapour pressure p_v_* at ambient temperature [16]. An estimate of the ratio of the number of moles of vapour to the total gas inside the bubble (i.e., *Π_2_*) can be obtained from the ideal gas equation of state as

(31) $$\frac{n_{w}}{n_{t}}=\frac{p_{v}^{*}}{P_{E}}$$

## 4 Results and discussion

### 4.1 Sonochemical activity measurements

**Fig. 3:**
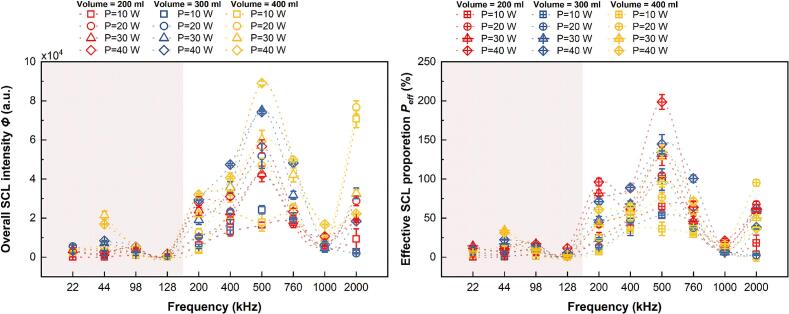
Sonochemistry measured by SCL activity expressed as (a) the overall SCL intensity (*Φ*) and (b) the effective SCL proportion (*P*_eff_)*.*

**Fig. 4:**
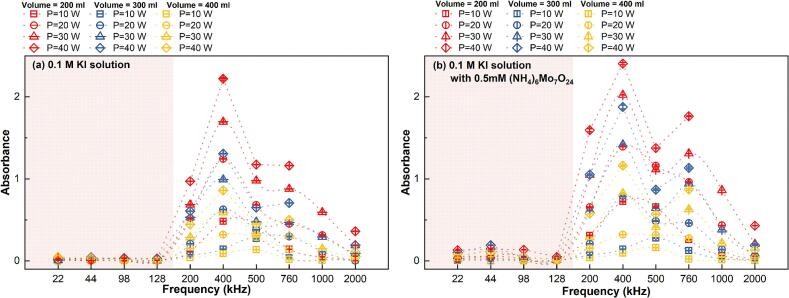
Sonochemistry measured by KI dosimetry with different testing solution: (a) 0.1 M KI solution and (b) 0.1 M KI solution with 0.5 mM catalyst.

Although SCL and KI dosimetry are sonochemical reaction processes, it is essential to address each specifically due to their distinct chemical mechanisms and reaction locations [8]. In our previous work [39], the results derived from the two different measurements of sonochemical activity were reported in dimensional forms. Herein, full dimensionless processing was adopted to maintain consistency in model structure and eliminate unit-dependence. This enables reliable regression analysis and supports broader applicability across different systems. The corresponding dimensionless outcomes, e.g., intensity *Φ* (via Eq. (6) and effective proportion *P*_eff_ (via Eq. (7) for SCL, and absorbance values from iodide dosimetry are presented in Fig. 3, Fig. 4.

In the SCL measurement, overall SCL intensity (*Φ*) and effective SCL proportion (*P*_eff_*)* exhibit divergent trends across different reactor volumes (Fig. 3). An increased liquid height or a larger reactor volume (yellow) results in an increased luminous intensity, yet the ratio of the luminous area to the total reactor’s cross-section decreases. Distinct variations in sonochemical activities with respect to frequency were observed above and below 128 kHz. In the lower frequency range (22 kHz ≤ *f* ≤ 128 kHz), both SCL outcomes exhibit lower outputs. Conversely, above 128 kHz, specifically between 200 and 760 kHz in our configuration, both SCL measurements increase. When the frequency reaches 1000 kHz, the SCL activity exhibits a significant decrease, with both the overall SCL intensity (*Φ*) and effective SCL proportion (*P_eff_)* dropping to lower levels. Beyond this point, a clear inflexion point emerges, marked by a shift in the trend—SCL activity transitions from decreasing to increasing as the frequency continues to rise.

It is worth noting that *P*_eff_ values can exceed 100 % in some configurations. This parameter represents the proportion of the luminescent area relative to the reactor (liquid) cross-section [39]. This occurs under several mid-frequency (300-760 kHz) acoustic conditions. Radiation forces in a travelling wave field can drive cavitation bubbles toward the liquid surface, deforming the air–liquid interface. High intensities may produce a fountain-like effect, concentrating light emission beyond the nominal surface area. Similar surface deformation phenomena, such as droplet ejection and fountain-like protrusions induced by acoustic radiation pressure, have also been observed in some ultrasonic systems [60]. Therefore, the observed *P*_eff_ values above 100 % are consistent with known acoustic effects and should be regarded as physically meaningful, not anomalous.

In KI dosimetry, with and without the catalyst, the raw data collected through UV–Vis is absorbance is dimensionless. Normally the absorbance is converted to concentration via the Beer-Lambert law, however for use in this analysis absorbance data was used directly (Fig. 4.). Absorbance levels indicate the production of I_3_^−^ during the sonication, namely the oxidation from sonochemical reactions. In a KI solution without the catalyst the I_3_^−^ production indicates the yield of iodide oxidising radicals (IORS) whereas the solution with the catalyst indicates the yield of IORS and H_2_O_2_ from sonication. Similar to SCL measurements, there is a switch in activity levels above and below 128 kHz (Fig. 4).

Overall, the SCL and KI measurement methods both indicate relatively low sonochemical activity within the 22–128 kHz frequency range. To capture the changing patterns across various frequency zones, the subsequent fittings were carried out using a piecewise approach i.e., 128 kHz was used as the boundary condition for the fitting process. The sonochemical activity increases significantly when the frequency reaches 200 kHz, marking the transition to a higher reactivity level. Given the apparent differences in sonochemical activity reflected by the SCL and KI methods around 1000 kHz, it is essential to handle data within this frequency range with caution. These discrepancies may reflect the complex changes in cavitation mechanisms that occur under high-frequency ultrasound conditions. Therefore, special attention should be paid to data accuracy when collecting and analysing results near this inflexion point.

**Fig. 5:**
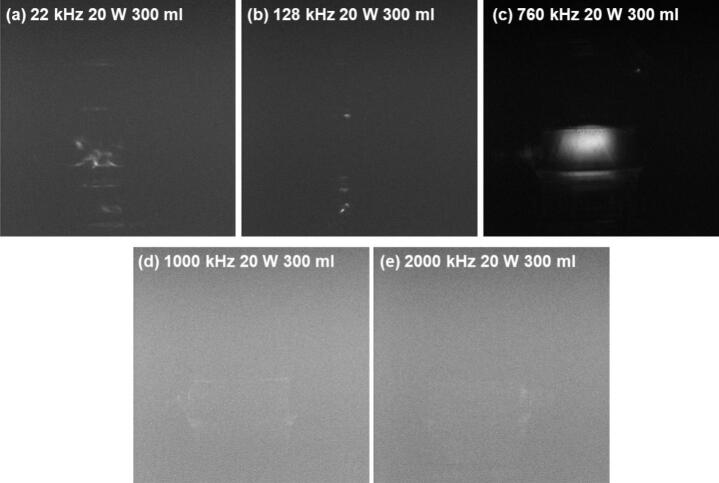
SCL emissions captured at various frequencies under 20 W power and 300 mL volume (critical frequencies: 22, 128, 1000, and 2000 kHz; control frequency: 760 kHz).

Regarding data reliability, KI dosimetry relies on UV–Vis spectroscopy to measure the absorbance of triiodide ions (I_3_^−^) generated during the formation of hydroxyl radicals [9,12]. This method offers strong objectivity, high repeatability, and quantitative accuracy, providing reliable support for evaluating the amount of free radicals generated. Thus, KI measurements can be considered a highly accurate reference. In contrast, SCL measurement depends on image acquisition and subsequent processing to extract the overall intensity and effective area proportion of SCL. This process involves image processing algorithms, lighting conditions, and background noise, which may introduce systematic errors or subjective bias during data collection [61]. These potential discrepancies become particularly critical near inflection points, so the raw images from which we derived the SCL activity inflection points were carefully analysed to ensure the accuracy of subsequent quantitative work. A set of SCL images at the critical frequency for the representative condition of 300 mL at 20 W is presented in Fig. 5. SCL images corresponding to other liquid heights and power settings at the critical frequencies are provided in the Appendix C (Fig. S3).

As shown in Fig. 5, the quality of SCL emission images at low and medium frequencies (22–760 kHz) is significantly higher than those captured at higher frequencies (1000 kHz and 2000 kHz). High-quality images enable more reliable quantitative analysis by facilitating accurate localization of active SCL regions and statistical evaluation of grayscale values. The poor image quality at ultra-high frequencies can be attributed to multiple physical and technical factors. Firstly, cavitation bubbles at such high frequencies are typically smaller and collapse less violently, further reducing the SCL signal intensity [8]. Secondly, repeated transitions between vaporous and gaseous cavitation are known to occur under ultrahigh-frequency irradiation [62], resulting in unstable and intermittent light emission. Additionally, in order to capture weak emissions, the imaging system requires increased sensitivity, which inevitably amplifies background noise [39]. These combined effects hinder the reliable identification of luminescent regions (Figs. 5 and S1), and may compromise the accuracy of subsequent quantification. In particular, it becomes difficult to distinguish actual SCL signals from noise under high background noise levels, making it challenging to define consistent luminescent boundaries. As a result, the data extracted from such images may be unreliable for quantitative modelling. In addition, 1000 kHz and 2000 kHz fall outside the two primary frequency segments used for parametric regression (22–128 kHz and 200–760 kHz, Fig. 3), and the limited number of valid points in this range further constrained their suitability for modelling. Therefore, SCL images at these ultra-high frequencies were excluded from the modelling analysis.

### 4.2 Physically-driven empirical model in sonochemical activities

According to the theoretical review presented in Section 3, a total of 12 parameters related to sonochemistry are summarized in Table 1. Based on Buckingham’s *Pi* theorem, 7 dimensionless *Π* terms were derived from these parameters (Table 3). The relationship between these dimensionless terms and sonochemical activity is investigated. Additionally, multivariate linear regression is applied to quantify this relationship and develop empirical correlations.

Nonlinear attributes of the actual acoustic system [60], resulting from intricate parameter influences and bubble dynamics, are considered. A logarithmic (ln) transformation is applied to approximate and linearize the intricate nonlinear relationship, enhancing the model's accuracy and predictive capability. Furthermore, the sonochemical activity index is denoted as sonochemical activity (SCA) which includes *Φ*, *P_eff_* and absorbance in this work. The fundamental correlation between sonochemical activities and the dimensionless values derived from the theoretical review can be established as (Eq. (32):

(32) $$\mathrm{SCA}=K{\left(\Pi 1\right)}^{x_{1}}{\left(\Pi 2\right)}^{x_{2}}{\left(\Pi 3\right)}^{x_{3}}{\left(\Pi 4\right)}^{x_{4}}{\left(\Pi 5\right)}^{x_{5}}{\left(\Pi 6\right)}^{x_{6}}{\left(\Pi 7\right)}^{x_{7}}$$

The logarithmic processing produces (Eq. (33):

(33) $$\begin{array}{c} \ln\left(\mathrm{SCA}\right)=\ln K+x_{1}\ln\left(\Pi 1\right)+x_{2}\ln\left(\Pi 2\right)+x_{3}\ln\left(\Pi 3\right)+x_{4}\ln\left(\Pi 4\right)+\\ x_{5}\ln\left(\Pi 5\right)+x_{6}\ln\left(\Pi 6\right)+x_{7}\ln\left(\Pi 7\right) \end{array}$$

An interpretation of the physical meaning of these dimensionless groups will be provided in Section 4.3.2, following the modelling and validation outcomes.

#### 4.2.1 Physically-driven empirical model of SCL

##### 4.2.1.1 Modelling of the overall SCL intensity (Φ)

The variation trend of the original value of the total luminescence intensity (*Φ*) with different dimensionless numbers under all test conditions is shown in Appendix D (Fig. S4). The highly erratic variances indicate the non-linear impacts on the system's sonochemistry response. Elementary linear correlations are unable to capture the complicated link between sonochemical activity and several relevant dimensionless numbers in ultrasonic processing.

To establish the quantitative relationships between the overall SCL intensity (*Φ*) and the dimensionless parameters across different frequency ranges, multivariable linear regression (based on logarithmic transformation) was performed in two distinct frequency zones: low-frequency (22–128 kHz) and mid-frequency (200–760 kHz). The resulting regression coefficients are summarized in Table 4, Table 5, respectively, and serve as the basis for the following analysis. Regression results for higher frequencies (*f*≥1000 kHz) are presented in Fig. S5 and Table S2 (Appendix D). These data were excluded from the main analysis due to potential deviations in SCL behaviour at extreme frequencies [7] and poor image quality, which reduced the accuracy of quantification.

**Table 5:** Results of the MLR analysis about *Φ* at 22 kHz *≤ f ≤* 128 kHz.

Parameter	Correlation coefficient	Parameter	Correlation coefficient	R^2^
ln*K*	5665.49447	*x4*	1.46771	0.7121
*x1*	−1110.65356	*x5*	871.39831	
*x2*	103.53056	*x6*	1108.31392	
*x3*	9686.80352	*x7*	0.7121	

**Fig. 6:**
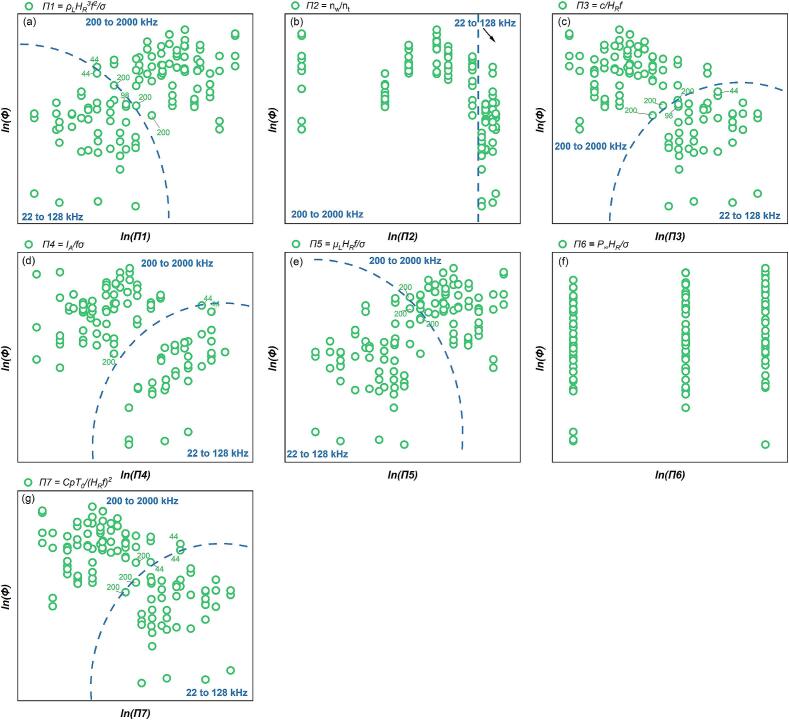
Trend of overall SCL intensity, *Φ* versus each dimensionless number: (a)*Π1,* (b) *Π2,* (c) *Π3,* (d) *Π4,* (e) *Π5,* (f) *Π6* and (g) *Π7* (logarithmic translation version, the frequency (kHz) of boundary data is indicated in blue).

**Fig. 7:**
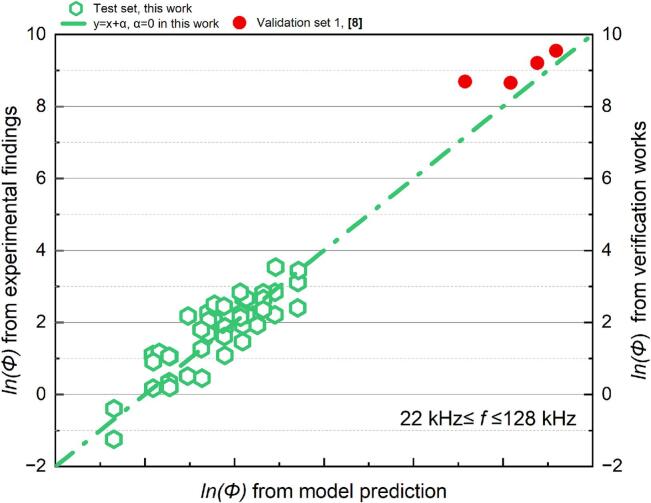
Comparison of the experimental and predicted results about *Φ* (22 kHz *≤ f ≤* 128 kHz): test set in this work (green) and validation set (red).

As shown in Fig. 6, logarithmic transformation enhances the visualization of overall SCL intensity Φ trends. Fig. 6(a) and (e) demonstrate that an increase in *Π1* and *Π5* increases the *Φ* value. Fig. 6(c) and (g) indicate that an elevation in *Π3* and *Π7* will reduce the *Φ* value. Across all experimental settings (22-2000 kHz), *Φ* tends to decrease as *Π4* increases. This indicates that higher *Π4* values correspond to lower total luminous intensity in the low-frequency range. However, in the individual frequency zones, i.e. *f* ≤ 128 kHz and *f* ≥ 200 kHz, *Φ* increases as the *Π4* value rises (Fig. 6(d)). Considering the centralization features of the data distribution (Fig. 6), and the relationship between SCL activity and individual variables (Fig. 3(a)), the multivariate linear regression fitting is initially divided into two distinct frequency domains: a low-frequency domain (*f*≤128 kHz) and a high-frequency domain (*f*≥200 kHz), as indicated by the dashed lines in Fig. 6. This division aims to establish a clearer relationship between multifactorial influences and *Φ* within each frequency range. The coefficients representing the relationship between the independent and dependent variables, as determined by multiple regression analysis in the two zones, are presented in Table 5, Table 6. The consistency between the experimental and predicted values in different frequency ranges are shown in Fig. 7 (*f* ≤ 128 kHz) and 8 (200 kHz ≤ *f* ≤ 760 kHz), respectively.

**Table 6:** Results of the MLR analysis about *Φ* at 200 kHz *≤ f ≤* 760 kHz.

Parameter	Correlation coefficient	Parameter	Correlation coefficient	R^2^
ln*K*	−149361.07091	*x4*	0.85027	0.8339
*x1*	20943.54867	*x5*	−11274.22676	
*x2*	17.65765	*x6*	−20947.14477	
*x3*	−57302.4901	*x7*	43955.61225

**Fig. 8:**
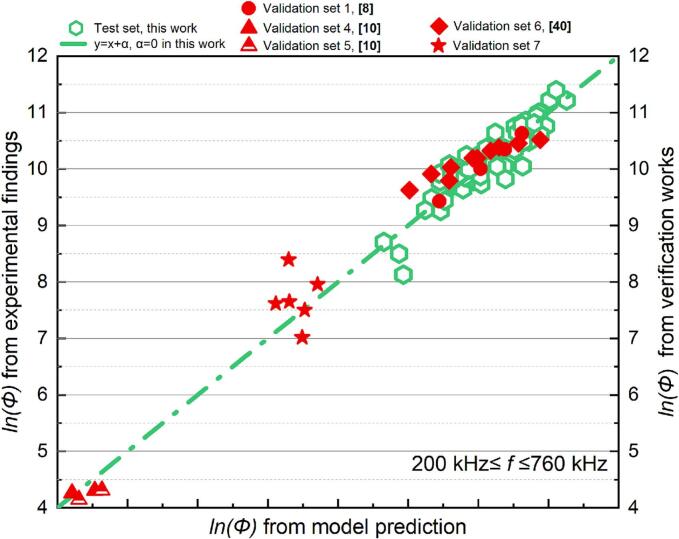
Comparison of the experimental and predicted results about *Φ* (200 kHz *≤ f ≤* 760 kHz): test set in this work (green) and verification set (red).

As shown in Fig. 7, Fig. 8, the models for the two frequency ranges are evaluated by comparing measured values to anticipated values (in red). In the comparison procedure, a constant term α is added to model prediction values across validation sets. This could be interpreted as an adjustment for the variation with *lnK* in Eq. (33) across different experimental batches. The correction term derived from the test set from this work is not universally applicable to all random test sets.

The proposed model struggles to accurately predict the value of sonochemical activity attributed to the high sensitivity of cavitation [7,21]. Along with cavitation's core physical properties, some subtle changes that are easily overlooked might affect the system's reaction, requiring extra adjustments. During operation, minor degradation in piezoelectric ceramic materials can cause slight shifts in transducer properties, such as electrical admittance, resonant frequency, and impedance. These subtle changes can alter the spatial distribution of the acoustic field. Since sonochemical reactions are highly sensitive to local sound intensity and cavitation behaviour, even small differences in the acoustic field between experimental groups can lead to variations in reaction results [63]. Similarly container wall form and material impacts energy reflection and absorption during energy propagation [21,64]. Those potential considerations make regression analysis corrective term unification practically impossible. Each reactor's ultrasonic chemical response and usage duration may vary, requiring unique corrections.

In this work, the model's predictions primarily emphasise the overall trends in the experimental and predicted values. The validation will focus on evaluating the reliability of the dimensionless array developed during sonication and providing a theoretical basis for parameter preselection for configuring the acoustic system through the consistency of trends between experimental and predicted values. To follow the aforementioned objectives and to provide a more comprehensive discussion of the predictive efficacy of the proposed model across many tests, the α for each validation group is modified for graphical presentation. The original slope for each group is maintained, and the data points’ intercept is adjusted to enhance the observability of the model verification results.

The assessed SCL luminescence intensity levels of the validation sets exhibited a positive association with the expected values derived from the model and beginning conditions (Fig. 7, Fig. 8). The alignment between the measured and projected values was more accurately represented in the cylindrical reactor than cubic reactor (Fig. 8, star symbol). The SCL intensity of the reactor with various configurations can be preliminarily evaluated using the suggested model, serving as an inspiration for developing the acoustic process parameters in practical applications.

##### 4.2.1.2 Modelling of the effective SCL proportion (P_eff_)

**Fig. 9:**
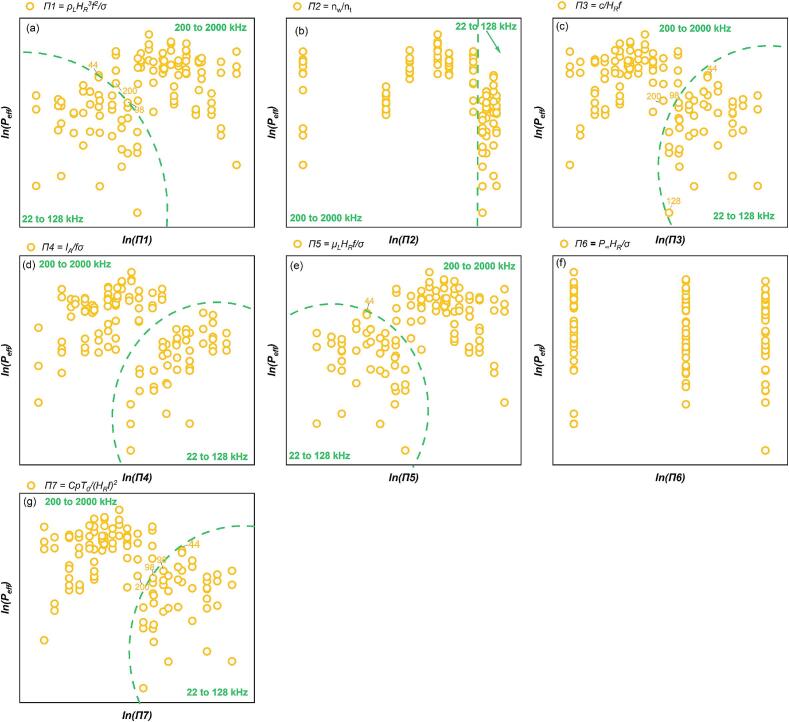
Trend of effective SCL proportion, *P_eff_* versus each dimensionless number from SCL experiment: (a)*Π1,* (b) *Π2,* (c) *Π3,* (d) *Π4,* (e) *Π5,* (f) *Π6* and (g) *Π7* (logarithmic translation version, the frequency (kHz) of boundary data is labelled).

Compared to the overall SCL intensity (*Φ)*, the outcome for the effective SCL proportion (*P_eff_*) is more standardised for an individual reactor. It removes the impact of volume discrepancies in experimental setup. The connection between *P_eff_* and each dimensionless value was analysed graphically. Fig. S6 (Appendix E) illustrates the intricate nonlinear correlation between the SCL response and each dimensionless parameter, namely the system parameters. Fig. 9 illustrates the relationship between the *P_eff_* and each dimensionless number after a logarithmic translation. The data distribution indicates that multiple regression must be conducted in at least two distinct frequency ranges: *f* ≤ 128 and 200 kHz ≤ *f*. The findings of the *P_eff_* likewise demonstrate the typical response of bubbles to SCL (Fig. 3). The correlation between *P_eff_* and the dimensionless values in the lower frequency range (*f* ≤ 128 kHz) is more inconsistent than in the higher frequency range. *P_eff_* merely shows a trend of increase corresponding to an increase in *Π4* value. Potential explanations for this phenomenon include reducing sonochemical activity when power increases beyond a critical intensity [13,53]. The increased size and collapse strength of the bubbles in low-frequency irradiation facilitates a more rapid attainment of this critical value. In the higher frequency zone, (200 to 2000 kHz), the *P_eff_* value diminishes with the increase of *Π1* and *Π5* (Fig. 9 (a) and (e)), whereas it increases with the increase of *Π3* and *Π7* (Fig. 9 (c) and (g)).

**Fig. 10:**
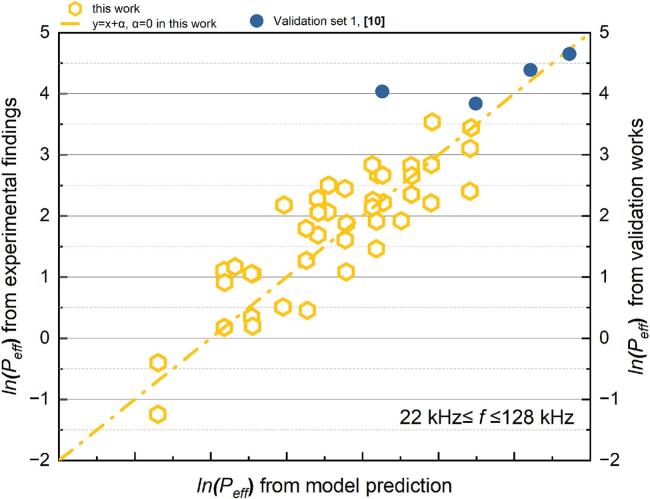
Comparison of the experimental and predicted results about *P_eff_* (22 kHz *≤ f ≤* 128 kHz): test set in this work (yellow) and verification set (blue).

**Fig. 11:**
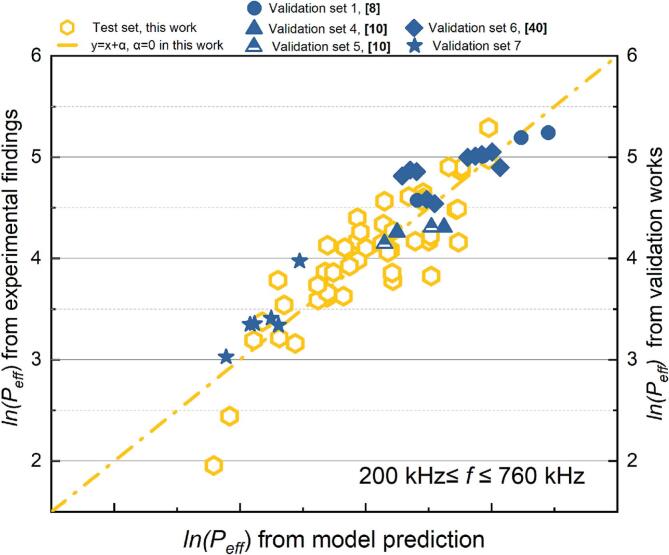
Comparison of the experimental and predicted results about *P_eff_* (200 kHz *≤ f ≤* 760 kHz): test set in this work (yellow) and verification set (blue).

Based on the previous discussion, SCL results obtained at frequencies exceeding 1000 kHz are not suitable for the regression analysis conducted in this study. The MLR analysis of the proportion of the active SCL area is predicated on the findings from the low frequency (22 kHz ≤ *f* ≤ 128 kHz) and medium frequency (200 kHz ≤ *f* ≤ 760 kHz) zones. The quantitative outcomes of the correlation in the two frequency ranges, *P_eff_*, and all dimensionless quantity, as obtained from the multiple linear regression analysis, are displayed in Table 7 (for 22 kHz ≤ *f* ≤ 128 kHz) and Table 8 (for 200 kHz ≤ *f* ≤ 760 kHz). The link between the predicted values and actual experimental findings in the two distinct frequency ranges is shown in Fig. 10 (22–128 kHz) and Fig. 11 (200–760 kHz), respectively.

**Table 7:** Results of the MLR analysis about *P_eff_* at 22 kHz *≤ f ≤* 128 kHz.

Parameter	Correlation coefficient	Parameter	Correlation coefficient	R^2^
ln*K*	144335.3283	*x4*	1.77841	0.71207
*x1*	−23013.49301	*x5*	24175.69222	
*x2*	125.90983	*x6*	23009.10169	
*x3*	22995.85877	*x7*	−22425.78306

**Table 8:** Results of the MLR analysis about *P_eff_* at 200 kHz *≤ f ≤* 760 kHz.

Parameter	Correlation coefficient	Parameter	Correlation coefficient	R^2^
ln*K*	−107976.40731	*x4*	0.75125	0.76789
*x1*	19245.28579	*x5*	−20229.1873	
*x2*	16.62405	*x6*	−19249.47594	
*x3*	−60880.97605	*x7*	39569.31993

*P_eff_* can be predicted using the relevant model with various initial configurations, as indicated by the results in the validation set. In assessing sonochemical activity in a cubic reactor (the star-marked data points in Fig. 8, Fig. 11), the evaluation of *P_eff_* provides results that are more favourable for parametric screening compared to the evaluation of *Φ*; mainly the predicted values within the group and the actual values exhibit greater consistency in their variations. This indicates that the assessment and forecasting of standardised items could better facilitate the generalisation of the proposed correlation between ultrasonic reactivity and initial settings across different reactors.

#### 4.2.2 Physically-driven empirical model of ultrasonic oxidation

##### 4.2.2.1 Modelling of the generation of IORS

**Fig. 12:**
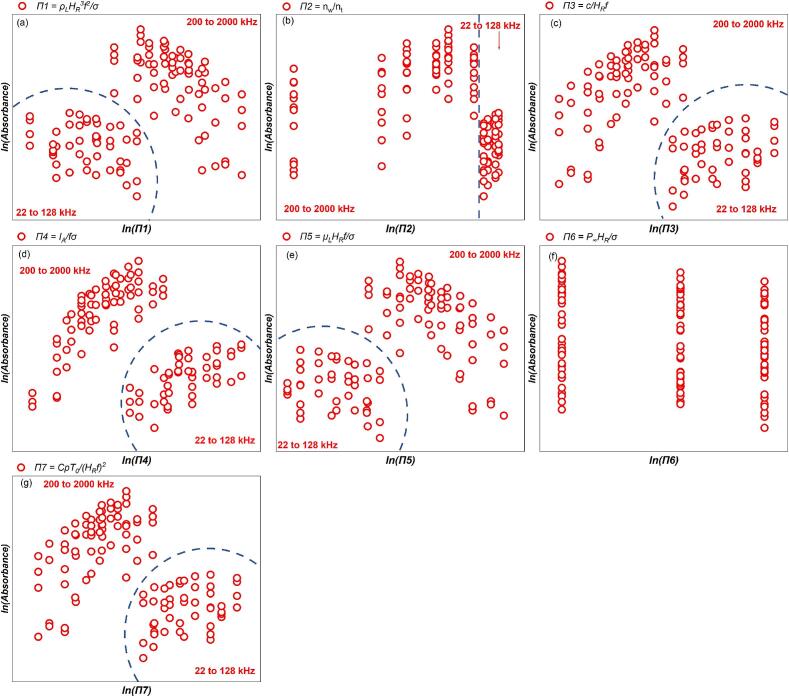
Trend of absorbance of KI solution after 10 min sonication, absorbance with each dimensionless number from dosimetry measurement: (a)*Π1,* (b) *Π2,* (c) *Π3,* (d) *Π4,* (e) *Π5,* (f) *Π6* and (g) *Π7* (logarithmic translation version).

The absorbance of sonicated KI solution reveals the generation of IORS. The correlation between the post-sonication absorbance and each dimensionless number after logarithmic transformation is shown in Fig. 12. Unlike the results observed in SCL measurements, the dosimetry results exhibit a higher degree of data concentration within specific frequency ranges (22–128 kHz and 200–2000 kHz), with no data points located near the boundaries of these frequency partitions. This clearer frequency distinction might arise because KI dosimetry captures the overall chemical activity throughout the entire reactor volume, particularly the cumulative generation of reactive oxygen species, which increases with frequency due to the more frequent collapse of cavitation bubbles [65]. In contrast, SCL primarily reflects the transient physical state of bubbles in the liquid during ultrasonic irradiation. Additionally, the global nature of KI dosimetry measurements, combined with their higher sensitivity and stability, minimizes the influence of localized effects and experimental noise. This, in turn, enhances the clarity of frequency-dependent bubble response characteristics.

**Fig. 13:**
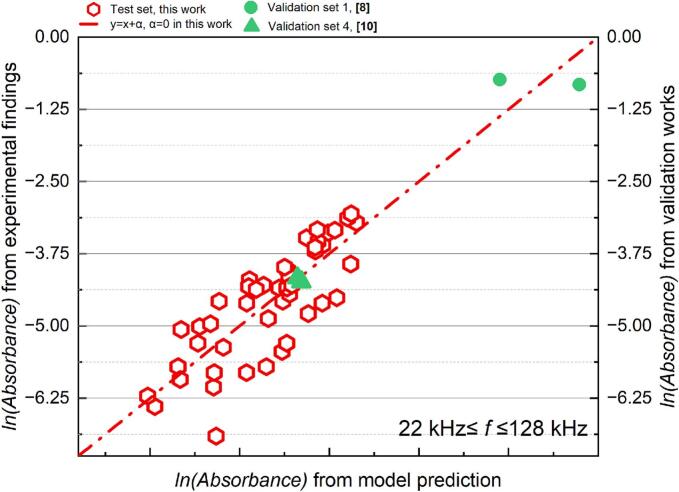
Comparison of the experimental and predicted results about absorbance of KI solution after sonication (22 kHz *≤ f ≤* 128 kHz): test set in this work (red) and validation set (green).

**Fig. 14:**
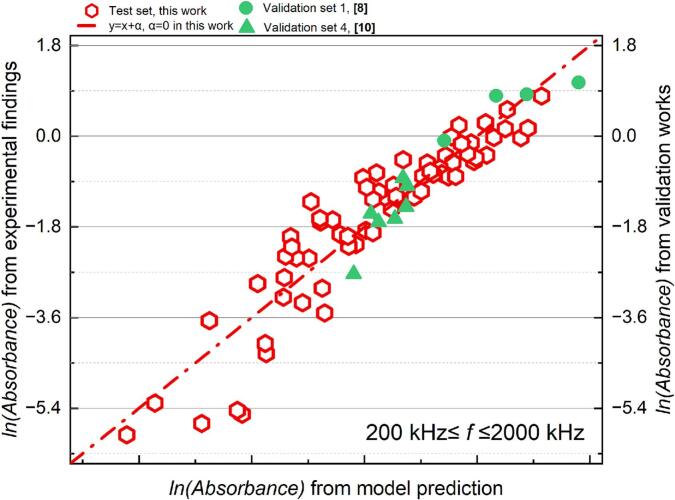
Comparison of the experimental and predicted results about absorbance of KI solution after sonication (200 kHz *≤ f ≤* 2000 kHz): test set in this work (red) and validation set (green).

Systems operating within the low frequency (*f* ≤ 128 kHz) have low absorbance values; in other words, these reactors demonstrate lower sonochemical oxidation. In the two distinct frequency ranges, sonochemical oxidative capacity escalates with increases in *Π3*, *Π4*, and *Π7* (Fig. 12 (c), (d) and (g)), whereas it diminishes with rises in *Π*1 and *Π5* (Fig. 12 (a) and (e). According to the characteristics of the dosimetry response of bubbles to sonochemistry at different frequencies (Fig. 4), the relationship between ultrasonically-induced oxidation and the group of dimensionless numbers was established in two frequency ranges: 22 to 128 kHz (Table. 9 and Fig. 13) and 200 to 2000 kHz (Table. 10 and Fig. 14).

**Table 9:** Results of the MLR analysis about absorbance of KI solution after sonication at 22 kHz *≤ f ≤* 128 kHz.

Parameter	Correlation coefficient	Parameter	Correlation coefficient	R^2^
ln*K*	199480.83111	*x4*	1.37941	0.67429
*x1*	−39624.79429	*x5*	52986.95139	
*x2*	33.10553	*x6*	39622.07102	
*x3*	99097.36814	*x7*	−62681.02872

**Table 10:** Results of the MLR analysis about absorbance of KI solution after sonication at 200 kHz *≤ f ≤* 2000 kHz.

Parameter	Correlation coefficient	Parameter	Correlation coefficient	R^2^
ln*K*	81154.11133	*x4*	1.90168	0.85929
*x1*	−8237.66572	*x5*	−4005.91987	
*x2*	11.20547	*x6*	8232.40101	
*x3*	24973.60389	*x7*	−22729.13803

In the low-frequency range (22–128 kHz), the MLR findings indicate that it is hard to elucidate the generation of IORS via the defined dimensionless quantities group (R^2^ = 0.674, Table. 8). The complicating elucidation of the IORS generation mechanism in this zone may result from the properties of bubbles in low-frequency systems. The larger critical sizes and prolonged expansion durations of bubbles lead IORS (especially ·OH) to engage in more erratic or intricate recombination reactions within the bubble before its rupture [66]. In low-frequency (*f* ≤ 128 kHz) tests, predicting acoustic oxidative activity using the sonochemical activity (ln*(*absorbance*)*) model proves challenging, as evidenced not only by the significant disparity between the predicted and actual values in the test set but also by the divergence between the predicted trends and actual variations in the validation set. (Fig. 13).

The predictive model based on the post-sonication absorbance of KI solution demonstrates strong applicability across different experimental sets, as evidenced by the consistent positive correlation between predicted values and experimental findings within the frequency range of 200 kHz ≤ *f* ≤ 2000 kHz (Fig. 14). Despite minor numerical discrepancies, this correlation across both the test and validation sets confirms the model’s ability to capture the underlying trends of IORS generation. Notably, the validation experiments conducted with identical geometric configurations—using the same plate-type transducer, reactor geometric configuration, and test solution composition—still show deviations attributed to the sensitivity of ultrasonic processes to environmental fluctuations (e.g., equipment status). Nevertheless, the model still effectively reflects sonochemical activity, as indicated by clustering test and validation data points along the ideal correlation line. This reliability makes the model a first step towards a practical tool for predicting IORS production and optimizing system parameters such as frequency, power input, and reactor geometry, ultimately reducing experimental workload while enhancing process efficiency.

##### 4.2.2.2 Modelling of the generation of IORS and H_2_O_2_

**Fig. 15:**
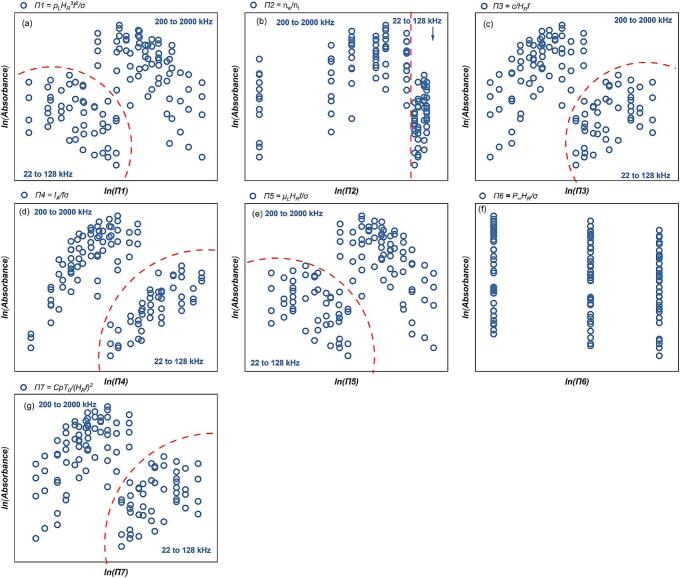
Trend of absorbance of catalyst-assisted KI solution after 10 min sonication, absorbance with each dimensionless number from dosimetry measurement: (a) *Π1,* (b) *Π2,* (c) *Π3,* (d) *Π4,* (e) *Π5,* (f) *Π6* and (g) *Π7* (logarithmic translation version).

**Fig. 16:**
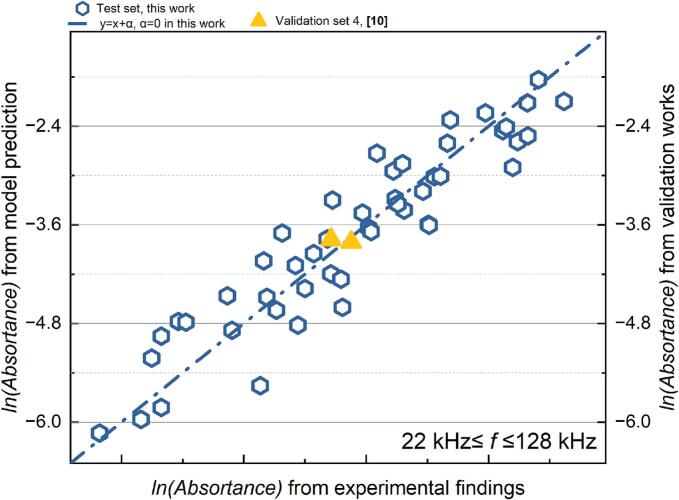
Comparison of the experimental and predicted results about absorbance of catalyst-assisted KI solution after sonication (22 kHz *≤ f ≤* 128 kHz): test set in this work (blue) and validation set (yellow).

**Fig. 17:**
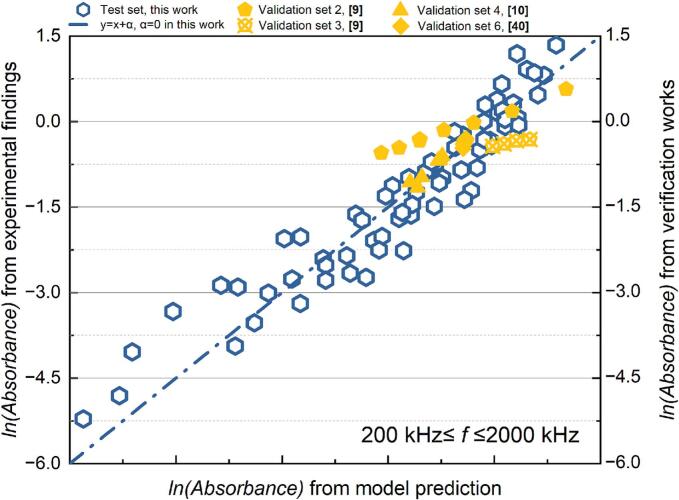
Comparison of the experimental and predicted results about absorbance of catalyst-assisted KI solution after sonication (200 kHz *≤ f ≤* 2000 kHz): test set in this work (blue) and validation set (yellow).

A KI solution with the ammonium molybdate catalyst which measures the total reactive oxygen species with H_2_O_2_ and the IORS [10]. The absorbance of catalyst-assisted KI solution was measured after 10 min ultrasonic irradiation. The sonochemical oxidation capacity exhibits a similar correlation with the dimensionless quantities, both with and without considering H_2_O_2_ production (Fig. 12, Fig. 15). Acoustic systems with higher *Π3*, *Π4*, and *Π7* show improved oxidation of iodine ions (Fig. 15 (c), (d) and (g)), while higher *Π1* and *Π5* correlate with reduced triiodide ion production (Fig. 15 (a) and (e)). This analogous trend for the H_2_O_2_ + IORS and just IORS is attributable, firstly, to the similar cavitation intensity/threshold within the system, which underpins sonochemical oxidation, and secondly, to the fact that the IORS produced by ultrasonic irradiation of a KI solution constitutes the primary source of free radicals with oxidative potential generated by ultrasonic irradiation [39]. The multivariate regression analysis was used to study the quantitative relationship between dimensionless quantities and ultrasonic oxidisability across two frequency ranges (Table 11: 22–128 kHz, Table 12: 200–2000 kHz). Fig. 16, Fig. 17 illustrate the correlation between actual and empirical model-predicted values across various frequency zones.

**Table 11:** Results of the MLR analysis about absorbance of catalyst-assisted KI solution after sonication at 22 kHz ≤ *f* ≤ 128 kHz.

Parameter	Correlation coefficient	Parameter	Correlation coefficient	R^2^
ln*K*	134986.84229	*x4*	1.70586	0.86872
*x1*	−45721.05887	*x5*	96532.27947	
*x2*	91.65213	*x6*	45716.75219	
*x3*	111075.10975	*x7*	−52994.21052

**Table 12:** Results of the MLR analysis about absorbance of catalyst-assisted KI solution after sonication at 200 kHz *≤ f ≤* 2000 kHz.

Parameter	Correlation coefficient	Parameter	Correlation coefficient	R^2^
ln*K*	61081.59905	*x4*	1.84337	0.90014
*x1*	−13400.91816	*x5*	19419.88072	
*x2*	12.40079	*x6*	13395.34803	
*x3*	41576.28951	*x7*	−24480.96531

In a low-frequency system (22–128 kHz), the established dimensionless group exhibits a stronger correlation with the system’s overall oxidizing capability—including the production of both H_2_O_2_ and IORS—compared to evaluating IORS generation alone. This is demonstrated by a higher determination coefficient for the combined production of H_2_O_2_ and IORS (R^2^(ln(*absorbance of the catalyst-assisted KI solution*)) = 0.869) than for IORS alone (R^2^(ln(*absorbance of the KI solution*)) = 0.674). H_2_O_2_, as a secondary reactive oxygen species (ROS) generated by free radical recombination, may exist in dynamic equilibrium with primary free radicals (·OH and ·H) [67]. Cavitation events related to the sonochemical reaction of H_2_O_2_ are presented in Eq. (34) to Eq. (41) [68]. Within low-frequency reactions, free radicals remain within the bubble for an extended duration, facilitating more recombination and breakdown, after which the bubble collapses, and secondary radicals are released·H_2_O_2_ constitutes a significant portion of the total free radicals released in these conditions. Thus, the complex ROS network may explain the reduced interpretive ability of the model devoid of H_2_O_2_.

(34) $$\cdot OH+\cdot OH\to H_{2}O_{2}$$

(35) $$HO_{2}\cdot +HO_{2}\cdot \to H_{2}O_{2}+O_{2}$$

(36) $$H\cdot +HO_{2}\cdot \to H_{2}O_{2}$$

(37) $$H_{2}O+\cdot OH\to H_{2}O_{2}+H\cdot$$

(38) $$2HO_{2}\to H_{2}O_{2}+O_{2}$$

(39) $$H\cdot +H_{2}O_{2}\to \cdot OH+H_{2}O$$

(40) $$H\cdot +H_{2}O_{2}\to H_{2}+HO_{2}\cdot$$

(41) $$\cdot OH+H_{2}O_{2}\to HO_{2}\cdot +H_{2}O$$

In the high-frequency range (200 kHz ≤ *f* ≤ 2000 kHz), the model demonstrates strong predictive accuracy for estimating the reactor's total oxidative capacity, including H_2_O_2_ and IORS production, with a high determination coefficient (R^2^ = 0.90). The evaluation results from the validation sets further confirm the model's robust predictive power (Fig. 17). Across different initial conditions, a consistent positive correlation between the actual sonochemical activity and predicted values indicates that the model effectively supports the pre-selection of process parameters.

In particular, the validation set includes two distinct reactor configurations. Validation sets 4 and 6 utilized the same cylindrical reactor as this work (inner diameter: 67 mm), while validation sets 2 and 3 used a smaller cylindrical reactor with an inner diameter of 50 mm. In validation sets 4 and 6, where the reactor configuration matched the test sets, the correlation between predicted and experimental values closely aligned with the test results, exhibiting a near-perfect relationship along a line with a slope of 1. This strong agreement underscores the model's reliability when applied to identical reactor configurations. Furthermore, the correlations observed in validation set 2 (which had a different liquid height) and validation set 3 (where the reaction solution was set at a different temperature) also demonstrated high consistency between predictions and experimental findings. This suggests that the regression model based on dimensionless parameters remains robust across various operating conditions. However, reactor geometry appears to be a critical factor for further improving the predictive model. This is evident from the consistent slope observed in the ratio of predicted to experimental values across validation sets 4 and 6, which share the same geometric configuration as the test set and validation sets 2 and 3. This consistency suggests that incorporating geometric corrections into the model could enhance its predictive accuracy across different reactor designs.

### 4.3 Insights and implications from the modelling process

In this study, seven dimensionless numbers (*Π1 – Π7*) were established based on Buckingham's *Pi* theorem to describe the relationship between sonochemical activity and initial operating parameters. The empirical summary of the modelling process and the comprehensive analysis of the prediction results help reveal the physical meaning behind the dimensionless numbers and further clarify the current model's scope of application and limitations. This section will discuss the following two aspects: (1) the insights of the empirical modelling process, and (2) a brief analysis of the physical meaning of the dimensionless numbers and their impact on sonochemical activity.

#### 4.3.1 Comparative analysis of the models

In this study, based on macroscopic trends observed (Fig. 3, Fig. 4) and the accuracy of the quantification method, multiple regression analysis was performed on SCL activity data in the 22–128 kHz and 200–760 kHz frequency ranges, while the multiple regression analysis of the KI measurements was performed in the 22–128 kHz and 200–2000 kHz frequency ranges. The models based on the KI measurements demonstrated greater accuracy in predicting the sonochemical activity, with much higher coefficients of determination (R^2^) for the regression models than for the models based on the SCL results. Specifically, in the 22–128 kHz range, the catalyst-assisted KI solution model achieved an R^2^ of 0.87, whereas the SCL-based models showed R^2^ values of 0.71 (*Φ*) and 0.77 (*P*_eff_), respectively. In the 200–760 kHz range, the R^2^ values for the SCL models were 0.82 (*Φ*) and 0.77 (*P*_eff_), while for the 200–2000 kHz range, the KI solution (without catalyst) model yielded an R^2^ of 0.86, and the catalyst-assisted KI solution achieved an even higher R^2^ of 0.90.

The quantification of SCL activity is fundamentally influenced by image quality and data processing procedures, which may introduce misleading noise and complicate the analysis of SCL activity [61]. In contrast, KI dosimetry, which relies on the cumulative chemical analysis of reactive oxygen species, is less vulnerable to transient fluctuations, resulting in more stable and consistent predictions. Additionally, the fundamental difference in measurement aims between SCL and KI dosimetry may lead to varying prediction performance. SCL reflects the transient luminescence at the gas–liquid interface of active cavitation bubbles, indicating the transient cavitation dynamics [8]. In contrast, KI dosimetry monitors the cumulative creation of reactive oxygen species, including IORS and H_2_O_2_, which offers a more integrated representation of the entire sonochemical activity [9].

Further analysis of the model performance across different frequency ranges revealed that regardless of the measurement method, the predictive accuracy of the regression models was consistently higher in the mid-to-high frequency range (200–760 kHz or 200–2000 kHz) compared to the low-frequency range (22–128 kHz). Specifically, the SCL-based model performed better in 200–760 kHz than in 22–128 kHz, whilst the KI-based model displayed significantly increased prediction accuracy in 200–2000 kHz compared to the low-frequency range (22–128 kHz). These findings provide theoretical support for the hypothesis that mid-to-high frequency ultrasound is more conducive to stable sonochemical reactions [21].

Interestingly, a substantial difference was seen between the two KI dosimetry approaches (IORS vs IORS + H_2_O_2_) in the low-frequency region (22–128 kHz), where R^2^ (post-sonication absorbance of KI solution) = 0.674 was notably lower than R^2^(post-sonication absorbance of KI solution with catalyst) = 0.869. Our previous work showed that bubble dynamic characteristics in low-frequencies enhance the generation of H_2_O_2_, increasing its proportion in the overall reactive oxygen species pool [39]. As a result, omitting H_2_O_2_ and evaluating only IORS may lead to an incomplete estimate of the sonochemical activity in low-frequency systems. This points out the necessity of considering the dynamic equilibrium of total reactive oxygen species in sonochemical systems instead of concentrating on individual species.

The KI dosimetry-based models displayed greater accuracy in predicting sonochemical activity than the SCL-based models. In particular, the catalyst-assisted measurement enabled a more comprehensive evaluation of the ultrasonic oxidation of H_2_O_2_ and IORS under various ultrasonic conditions. This approach also provided a more stable predictive framework for sonochemical activity in complete ultrasonic systems. However, due to the fundamental differences between SCL and KI dosimetry in measurement principles, target chemical processes, and the spatial localization of reactions within the system [8], both models hold significant value for experimental optimization, theoretical guidance, and the design of optimized sonochemical reaction parameters.

Compared to previous cavitation modelling studies, including the acoustic cavitation number models [33,38], the modelling scope was extended in this work. Earlier models primarily aimed to characterize physical cavitation phenomena, bubble cloud size or erosion potential. This study focuses on quantitatively predicting sonochemical activity using two representative measurement techniques: SCL analysis and KI dosimetry. Furthermore, a broader range of physical effects was integrated through the formulation of seven dimensionless parameters (*Π1*–*Π7*), which encompass the influences of acoustic, thermal, and chemical kinetics. Consequently, this framework could advance the ultrasonic dimensionless modelling approach toward chemically relevant and extended application situations.

#### 4.3.2 Physical interpretation of the dimensionless numbers

Based on theoretical review and Buckingham's *Pi* theorem, this study defines seven dimensionless numbers *(Π1*–*Π7*) to quantitatively describe the relationship between sonochemical activity and key physical parameters. However, due to cavitation's highly complex nature, fully elucidating these parameters' mechanistic roles remains challenging. Therefore, the discussion of dimensionless numbers in this study is primarily based on the modelling process and theoretical analysis, attempting to interpret their effects under different ultrasonic conditions.

According to the involved physical quantities and expressions, these dimensionless numbers can be categorised into four major groups:

(1) Bubble dynamics-related dimensionless numbers *(Π1, Π4, Π5*) which describe bubble formation, oscillation, and collapse. Bubble dynamics directly determine the intensity of cavitation, thereby influencing free radical generation and sonochemical activity [16].
(2) Cavitation environment-regulating dimensionless numbers (*Π2, Π6*), which reflect the effects of ambient pressure and bubble gas composition on cavitation intensity. The vapour content inside bubbles influences the potential for pyrolytic reactions and plays a buffering role in stabilising bubble temperature [58]. Previous studies have shown that bubble number and size are influenced by ambient pressure [69,70].
(3) Acoustic wave transfer and resonance-related dimensionless number (*Π3*) characterise ultrasound wave propagation and acoustic field distribution within the reaction system [56].
(4) Thermal effect-related dimensionless number (*Π7*) describes heat distribution during acoustic cavitation. Solutions with higher heat capacity exhibit greater thermal buffering capability [13], which may reduce local temperature fluctuations and consequently influence sonochemical activity.

The experimental results in Fig. 6, Fig. 9, Fig. 12, Fig. 15 demonstrate distinct trends for the defined dimensionless groups, aligning with their physical interpretations. In the dynamics group, *Π4* shows a strong positive correlation with sonochemical activity, confirming the importance of energy delivery. In contrast, *Π1* and *Π5* exhibit negative trends, which might be due to the dominating influence of frequency and viscosity, which limit bubble growth and collapse efficiency. *Π3* displays a strong positive correlation, reflecting the critical role of acoustic resonance conditions in enhancing cavitation. *Π7*, related to thermal buffering, shows a positive trend, suggesting that moderate heat capacity and initial temperature favour stable energy accumulation. For *Π2* and *Π6*, the relevant variables (e.g., vapour content and ambient pressure) showed limited variation under our experimental conditions. Consequently, it is difficult to identify clear trends. These trend analyses support the mechanistic role of each *Π*-term and highlight the importance of considering multi-parameter synergy in predicting sonochemical reactivity.

These dimensionless numbers successfully reveal the coupling relationships among bubble dynamics, the cavitation environment, energy propagation, and thermal effects in sonochemical processes. However, due to the nonlinear nature of acoustic systems and the complex interactions among multiple factors, optimising a single parameter alone is unlikely to provide a comprehensive guidance of sonochemical activity [21]. For instance, ultrasonic frequency affects the number of bubbles and determines their size, collapse energy [7]. The effect of acoustic pressure amplitude (i.e., ultrasonic power) is influenced by the cavitation threshold effect [71,72]. It is also necessary to recognise that MLR and similar statistical methods are subject to inherent limitations, particularly when the model structure lacks a solid foundation in physical theory. Apparent statistical correlations may arise without genuine physical causality, potentially leading to misleading interpretations [73]. The modelling framework was developed based on a theoretical review of bubble dynamics, reaction kinetics, and other proven physical parameters (section 3). Logarithmic transformation was also used to linearise nonlinear relationships. These measures help ensure physical consistency. Nevertheless, the risk of non-physical spurious correlations cannot be entirely excluded. Some observed correlations may still reflect statistical association rather than strict physical causality, particularly when a dominant factor influences multiple dependent variables. Frequency, for instance, is known to affect cavitation behaviour and acoustic wave propagation simultaneously [60,65], a common-cause effect that complicates the complete elimination of spurious correlations. This type of common-cause effect is often observed in ultrasonic systems [7,21], complicating the complete removal of non-physical false correlations. Therefore, the interpretation and optimisation of sonochemical performance based on dimensionless analysis must be conducted with caution, considering experimental validation and the synergistic effects of multiple factors under specific operating conditions.

In this study, the dimensionless modelling framework was developed and validated using experimental data obtained from a static, flat-plate ultrasonic reactor operating with aqueous solutions. Under these conditions, the defined dimensionless group demonstrated strong predictive capability for sonochemical activity. However, the applicability of this framework to other systems, such as non-aqueous solvents, probe-based reactors, or systems involving fluid motion, remains to be tested. If the proposed framework needs to be strengthened or adapted for specific operating conditions, broader data collection and a reassessment of key variables may be required. Once additional parameters (e.g., flow-related quantities) are confirmed to be influential, they can be incorporated into the prediction framework. This process would involve redefining the dimensionless groups and recalibrating the model to reflect updated quantitative relationships within the specific system. The refitting should be based on an extended dataset that reflects the influence of the added parameters.

## 5 Conclusions

This study systematically investigated the effects of ultrasonic parameters, solution properties, experimental environment, and reactor design on sonochemical activity. Seven key dimensionless numbers (*Π1*–*Π7*) were identified, providing insights into the roles of bubble dynamics, cavitation environment, acoustic wave propagation, and thermal effects in governing sonochemical processes. Through this analysis, a dimensionless multivariate regression model frame was developed to predict sonochemical activity under varying operating conditions. The results indicate that SCL and KI dosimetry measurements exhibit significant frequency dependence, with distinct activity patterns in the low-frequency range (22–128 kHz) and the medium-to-high frequency range (200–2000 kHz). The constructed dimensionless regression models accurately predict the trends of sonochemical activity under different operating conditions. Among these, KI dosimetry is a more reliable quantitative assessment tool for sonochemical activity than SCL measurements, as it quantifies the cumulative generation of reactive oxygen species and is less susceptible to experimental uncertainties.

The dimensionless analysis approach not only elucidates the multi-parameter coupling mechanisms influencing sonochemical activity but also serves as an effective theoretical framework for optimizing the design and operating conditions of ultrasonic reactors. This methodology enables the pre-experimental prediction of sonochemical activity, enhancing experimental efficiency while reducing optimization costs. Future research should extend this approach to non-aqueous systems, complex solution environments, and different ultrasonic reactor configurations. Additionally, integrating nonlinear analysis methods, such as machine learning or advanced regression models, could further refine the predictive accuracy of the model, enhancing its practical applicability and scalability in sonochemical research and industrial applications.

## CRediT authorship contribution statement

**Yucheng Zhu:** Writing – review & editing, Writing – original draft, Visualization, Validation, Methodology, Investigation, Formal analysis, Data curation, Conceptualization. **Xueliang Zhu:** Visualization, Methodology, Data curation. **Irem Soyler:** Investigation, Data curation. **Xuhai Pan:** Writing – review & editing, Supervision, Resources. **Lian X. Liu:** Writing – review & editing, Supervision. **Madeleine J. Bussemaker:** Writing – review & editing, Supervision, Resources, Project administration, Formal analysis, Conceptualization.

## Declaration of competing interest

The authors declare that they have no known competing financial interests or personal relationships that could have appeared to influence the work reported in this paper.

## References

[1] Yasui K. (2023). The reducing agents in sonochemical reactions without any additives. Molecules.

[2] Sidnell T., Wood R.J., Hurst J., Lee J., Bussemaker M.J. (2022). Sonolysis of per-and poly fluoroalkyl substances (PFAS): a meta-analysis. Ultrason. Sonochem..

[3] Bussemaker M.J., Xu F., Zhang D. (2013). Manipulation of ultrasonic effects on lignocellulose by varying the frequency, particle size, loading and stirring. Bioresour. Technol..

[4] Flores E.M., Cravotto G., Bizzi C.A., Santos D., Iop G.D. (2021). Ultrasound-assisted biomass valorization to industrial interesting products: state-of-the-art, perspectives and challenges. Ultrason. Sonochem..

[5] Khan N.A., Jhung S.H. (2015). Synthesis of metal-organic frameworks (MOFs) with microwave or ultrasound: Rapid reaction, phase-selectivity, and size reduction. Coord. Chem. Rev..

[6] Pandis P.K., Kalogirou C., Kanellou E., Vaitsis C., Savvidou M.G., Sourkouni G., Zorpas A.A., Argirusis C. (2022). Key points of advanced oxidation processes (AOPs) for wastewater, organic pollutants and pharmaceutical waste treatment: a mini review. ChemEngineering.

[7] Wood R.J., Lee J., Bussemaker M.J. (2017). A parametric review of sonochemistry: Control and augmentation of sonochemical activity in aqueous solutions. Ultrason. Sonochem..

[8] Wood R.J., Lee J., Bussemaker M.J. (2019). Disparities between sonoluminescence, sonochemiluminescence and dosimetry with frequency variation under flow. Ultrason. Sonochem..

[9] Merouani S., Hamdaoui O., Saoudi F., Chiha M. (2010). Influence of experimental parameters on sonochemistry dosimetries: KI oxidation, Fricke reaction and H2O2 production. J. Hazard. Mater..

[10] Zare M., Alfonso-Muniozguren P., Bussemaker M.J., Sears P., Serna-Galvis E.A., Torres-Palma R.A., Lee J. (2023). A fundamental study on the degradation of paracetamol under single-and dual-frequency ultrasound. Ultrason. Sonochem..

[11] Amaniampong P.N., Jérôme F. (2020). Catalysis under ultrasonic irradiation: a sound synergy. Curr. Opin. Green Sustainable Chem..

[12] Asakura Y., Yasuda K. (2021). Frequency and power dependence of the sonochemical reaction. Ultrason. Sonochem..

[13] Schieppati D., Mohan M., Blais B., Fattahi K., Patience G.S., Simmons B.A., Singh S., Boffito D.C. (2024). Characterization of the acoustic cavitation in ionic liquids in a horn-type ultrasound reactor. Ultrason. Sonochem..

[14] Sojahrood A., Wegierak D., Haghi H., Karshfian R., Kolios M.C. (2019). A simple method to analyze the super-harmonic and ultra-harmonic behavior of the acoustically excited bubble oscillator. Ultrason. Sonochem..

[15] Ince N., Tezcanli G., Belen R., Apikyan İ.G. (2001). Ultrasound as a catalyzer of aqueous reaction systems: the state of the art and environmental applications. Appl Catal B.

[16] Kalmár C., Klapcsik K., Hegedűs F. (2020). Relationship between the radial dynamics and the chemical production of a harmonically driven spherical bubble. Ultrason. Sonochem..

[17] Shen L., Pang S., Zhong M., Sun Y., Qayum A., Liu Y., Rashid A., Xu B., Liang Q., Ma H. (2023). A comprehensive review of ultrasonic assisted extraction (UAE) for bioactive components: Principles, advantages, equipment, and combined technologies. Ultrason. Sonochem..

[18] Harada H., Ono Y. (2015). Improvement of the rate of sono-oxidation in the presence of CO2. Jpn. J. Appl. Phys..

[19] Kanthale P., Ashokkumar M., Grieser F. (2008). Sonoluminescence, sonochemistry (H2O2 yield) and bubble dynamics: frequency and power effects. Ultrason. Sonochem..

[20] Matula T.J. (1999). Inertial cavitation and single–bubble sonoluminescence, Philosophical transactions of the Royal Society of London. Series a: Mathem., Phys. Eng. Sci..

[21] Meroni D., Djellabi R., Ashokkumar M., Bianchi C.L., Boffito D.C. (2021). Sonoprocessing: from concepts to large-scale reactors. Chem. Rev..

[22] Leighton T. (2012).

[23] Asakura Y., Nishida T., Matsuoka T., Koda S. (2008). Effects of ultrasonic frequency and liquid height on sonochemical efficiency of large-scale sonochemical reactors. Ultrason. Sonochem..

[24] Thangavadivel K., Okitsu K., Owens G., Lesniewski P.J., Nishimura R. (2013). Influence of sonochemical reactor diameter and liquid height on methyl orange degradation under 200 kHz indirect sonication. J. Environ. Chem. Eng..

[25] Little C., El-Sharif M., Hepher M. (2007). The effect of solution level on calorific and dosimetric results in a 70 kHz tower type sonochemical reactor. Ultrason. Sonochem..

[26] Weinberg M.C. (1981). Surface tension effects in gas bubble dissolution and growth. Chem. Eng. Sci..

[27] B. Han, B. Yang, Z.-H. Shen, J. Lu, X.-W. Ni, Numerical Investigation of the Influences of Liquid Viscosity, Surface Tension and Initial Bubble Gas Content on the Dynamic Properties of a Laser-Induced Cavitation Bubble, Lasers in Engineering (Old City Publishing), 19 (2010). https://www.researchgate.net/publication/288158858_Numerical_Investigation_of_the_Influences_of_Liquid_Viscosity_Surface_Tension_and_Initial_Bubble_Gas_Content_on_the_Dynamic_Properties_of_a_Laser-Induced_Cavitation_Bubble.

[28] Iwai Y., Li S. (2003). Cavitation erosion in waters having different surface tensions. Wear.

[29] Gogate P.R., Wilhelm A.M., Pandit A.B. (2003). Some aspects of the design of sonochemical reactors. Ultrason. Sonochem..

[30] Yasui K., Towata A., Tuziuti T., Kozuka T., Kato K. (2011). Effect of static pressure on acoustic energy radiated by cavitation bubbles in viscous liquids under ultrasound. J. Acoust. Soc. Am..

[31] Flannigan D.J., Suslick K.S. (2010). Inertially confined plasma in an imploding bubble. Nat. Phys..

[32] Gogate P.R., Sutkar V.S., Pandit A.B. (2011). Sonochemical reactors: important design and scale up considerations with a special emphasis on heterogeneous systems. Chem. Eng. J..

[33] Kozmus G., Zevnik J., Hočevar M., Dular M., Petkovšek M. (2022). Characterization of cavitation under ultrasonic horn tip–proposition of an acoustic cavitation parameter. Ultrason. Sonochem..

[34] Gogate P.R. (2008). Cavitational reactors for process intensification of chemical processing applications: a critical review. Chem. Eng. Process..

[35] Wang Q., Ding L., Xue Z., Chen T., Pan X., Short M. (2024). Bubble plume dispersion from underwater gas leakage: an experimental and dimensionless modelling study. Appl. Ocean Res..

[36] Zhu X., Pan X., Mei Y., Ma J., Tang H., Zhu Y., Liu L.X., Jiang J., Chen T. (2023). Thermal nonequilibrium and mechanical forces induced breakup and droplet formation of superheated liquid jets under depressurized release. Appl. Therm. Eng..

[37] Khafajah H., Ali M.I.H., Thomas N., Janajreh I., Arafat H.A. (2022). Utilizing Buckingham Pi theorem and multiple regression analysis in scaling up direct contact membrane distillation processes. Desalination.

[38] Dular M., Petkovšek M. (2018). Cavitation erosion in liquid nitrogen. Wear.

[39] Zhu Y., Zhu X., Pan X.-H., Liu L., Bussemaker M. (2025). Correlation of sonochemical activities measured via dosimetry and an area-selective analysis of sono (chemi) luminescence. RSC Mechanochemistry.

[40] Bridgman P.W. (1922).

[41] Sidnell T., Hurst J., Lee J., Bussemaker M.J. (2024). Increasing efficiency and treatment volumes for sonolysis of per-and poly-fluorinated substances, applied to aqueous film-forming foam. Ultrason. Sonochem..

[42] Dehane A., Merouani S., Chibani A., Hamdaoui O., Yasui K., Ashokkumar M. (2022). Estimation of the number density of active cavitation bubbles in a sono-irradiated aqueous solution using a thermodynamic approach. Ultrasonics.

[43] J. Luo, Z. Fang, R.L. Smith, X. Qi, Fundamentals of acoustic cavitation in sonochemistry, 2015.

[44] Keller J.B., Miksis M. (1980). Bubble oscillations of large amplitude. J. Acoust. Soc. Am..

[45] Merouani S., Hamdaoui O., Rezgui Y., Guemini M. (2015). Computer simulation of chemical reactions occurring in collapsing acoustical bubble: dependence of free radicals production on operational conditions. Res. Chem. Intermed..

[46] Zhang Y.-N., Li X.-F., Guo Z.-Y., Zhang Y.-N. (2019). Interior non-uniformity of acoustically excited oscillating gas bubbles. J. Hydrodyn..

[47] Young F.R. (1976). Sonoluminescence from water containing dissolved gases. J. Acoust. Soc. Am..

[48] McNamara W.B., Didenko Y.T., Suslick K.S. (2003). Pressure during sonoluminescence. J. Phys. Chem. B.

[49] Kerboua K., Hamdaoui O. (2017). Computational study of state equation effect on single acoustic cavitation bubble’s phenomenon. Ultrason. Sonochem..

[50] Merouani S., Hamdaoui O., Rezgui Y., Guemini M. (2013). Effects of ultrasound frequency and acoustic amplitude on the size of sonochemically active bubbles–theoretical study. Ultrason. Sonochem..

[51] Toegel R., Lohse D. (2003). Phase diagrams for sonoluminescing bubbles: a comparison between experiment and theory. J. Chem. Phys..

[52] S. Fujikawa, M. Maerefat, A study of the molecular mechanism of vapour condensation, JSME international journal. Ser. 2, Fluids engineering, heat transfer, power, combustion, thermophysical properties, 33 (1990) 634-641. https://doi.org/10.1299/jsmeb1988.33.4_634.

[53] K.S. Suslick, The chemistry of ultrasound, 1994.

[54] Henglein A., Kormann C. (1985). Scavenging of OH radicals produced in the sonolysis of water. Int. J. Radiat. Biol. Relat. Stud. Phys. Chem. Med..

[55] Tuziuti T., Yasui K., Sivakumar M., Iida Y., Miyoshi N. (2005). Correlation between acoustic cavitation noise and yield enhancement of sonochemical reaction by particle addition. Chem. A Eur. J..

[56] Laborde J.-L., Bouyer C., Caltagirone J.-P., Gérard A. (1998). Acoustic cavitation field prediction at low and high frequency ultrasounds. Ultrasonics.

[57] Yunus A.C. (2010).

[58] Ashokkumar M. (2011). The characterization of acoustic cavitation bubbles–an overview. Ultrason. Sonochem..

[59] Lv L., Lou Z., Wan C. (2024). Theoretical estimation of sonochemical characteristics in a single cavitation bubble under various static pressure conditions. AIP Adv..

[60] Lee J., Ashokkumar M., Yasui K., Tuziuti T., Kozuka T., Towata A., Iida Y. (2011). Development and optimization of acoustic bubble structures at high frequencies. Ultrason. Sonochem..

[61] Tiong T.J., Chandesa T., Yap Y.H. (2017). Comparison of sonochemiluminescence images using image analysis techniques and identification of acoustic pressure fields via simulation. Ultrason. Sonochem..

[62] Yasui K. (2018).

[63] Hemsel T., Bornmann P., Morita T., Sondermann-Wölke C., Sextro W. (2016). Reliability analysis of ultrasonic power transducers. Arch. Appl. Mech..

[64] Capelo J., Galesio M., Felisberto G., Vaz C., Pessoa J.C. (2005). Micro-focused ultrasonic solid–liquid extraction (μFUSLE) combined with HPLC and fluorescence detection for PAHs determination in sediments: optimization and linking with the analytical minimalism concept. Talanta.

[65] Beckett M.A., Hua I. (2001). Impact of ultrasonic frequency on aqueous sonoluminescence and sonochemistry. Chem. A Eur. J..

[66] Sunartio D., Ashokkumar M., Grieser F. (2007). Study of the coalescence of acoustic bubbles as a function of frequency, power, and water-soluble additives. J. Am. Chem. Soc..

[67] S.K. Bhangu, M. Ashokkumar, Theory of sonochemistry, 2017. 10.1007/s41061-016-0054-y 27573408

[68] Adewuyi Y.G. (2001). Sonochemistry: environmental science and engineering applications. Ind. Eng. Chem. Res..

[69] Brett H., Jellinek H. (1956). Degradation of long‐chain molecules by ultrasonic waves. Part VI. effect of pressure. J. Polym. Sci..

[70] Merouani S., Dehane A., Hamdaoui O., Yasui K., Ashokkumar M. (2024). Review on the impacts of external pressure on sonochemistry. Ultrason. Sonochem..

[71] Dange P., Kulkarni A., Rathod V. (2015). Ultrasound assisted synthesis of methyl butyrate using heterogeneous catalyst. Ultrason. Sonochem..

[72] Khan N.R., Jadhav S.V., Rathod V.K. (2015). Lipase catalysed synthesis of cetyl oleate using ultrasound: optimisation and kinetic studies. Ultrason. Sonochem..

[73] Yasui K. (2024). Merits and demerits of machine learning of ferroelectric, flexoelectric, and electrolytic properties of ceramic materials. Materials.

