# Supplementary material for: Prediction of sonochemical activity based on dimensionless analysis and multivariate linear regression
Source: Ultrason Sonochem. 2025 Jun 12;120:107427. doi: 10.1016/j.ultsonch.2025.107427 (PMC12221647; doi:10.1016/j.ultsonch.2025.107427)
Supplement: Supplementary Data 1 [file mmc1.docx]

**Supplementary Information (SI)**

**Appendix A**

**Experimental configurations of validation sets**

**Table S1** Initial conditions of the experiment (validation sets)

|  |  | **Set 1** | **Set 2** | **Set 3** | **Set 4** | **Set 5** | **Set 6** | **Set 7** |
| --- | --- | --- | --- | --- | --- | --- | --- | --- |
| **Ultrasonic parameters** | Frequency (kHz) | 44, 300, 1000 | 300 | 300 | 22, 98, 200, 300, 400, 500, 780, 850, 1000 | 22, 98, 200, 300, 400, 500, 780, 850, 1000 | 410 | 500, 760 |
|  | Load power (W) | 20, 40 | 60 | 60 | 20 | 20 | 20, 25, 40, 50, 60, 80, 100 | 30 |
| **Reactor geometric configuration** | Solution volume (ml) | 400 | 100, 150, 200, 250, 300, 350, 400 | 300 | 400 | 400 | 200, 250, 300, 400, 500 | 350, 500, 750 |
|  | Reactor shape | cylinder | cylinder | cylinder | cylinder | cylinder | cylinder | cube |
|  | size of piezoelectric disk (diameter, mm) | 50 | 40 | 40 | 50 | 50 | 50 | 50 |
|  | Reactor size (inner diameter/ side length, mm) | 64 | 50 | 50 | 64 | 64 | 64 | 110 |
| **Additional conditions** | Initial temperature of solution (℃) | 25 | 25 | 25, 35, 45, 55 | 25 | 25 | 20 | 25 |
|  | Pressure (atm) | 1 | 1 | 1 | 1 | 1 | 1 | 1 |
|  | Dosimetry solution | 0.1 M KI | 0.1 M KI + 0.01M catalyst | 0.1 M KI + 0.01M catalyst | 0.1 M KI + 0.5 mM catalyst | 0.1 M KI + 0.5 mM catalyst | 0.1 M KI + 0.5 mM catalyst | - |
|  | top with(out) a horn | - | - | - | - | 5 cm | - | - |
| **Ref.** |  | **[8]** | **[9]** | **[9]** | **[10]** | **[10]** | **[40]** |  |

**Appendix B**

**Experimental information about a cubic reactor**


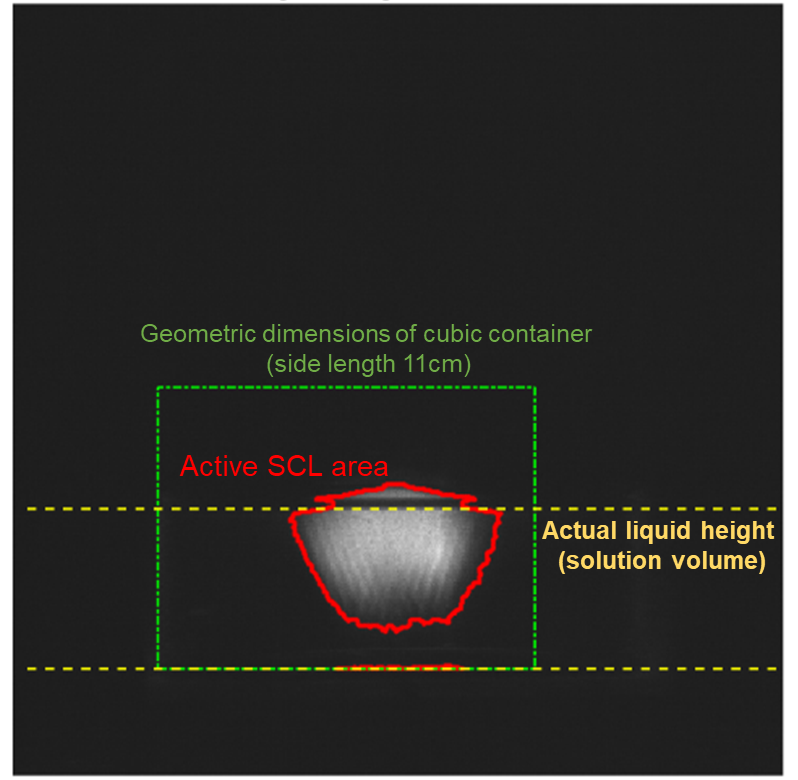


**Fig. S1** Schematic diagram of SCL analysis in a cubic reactor


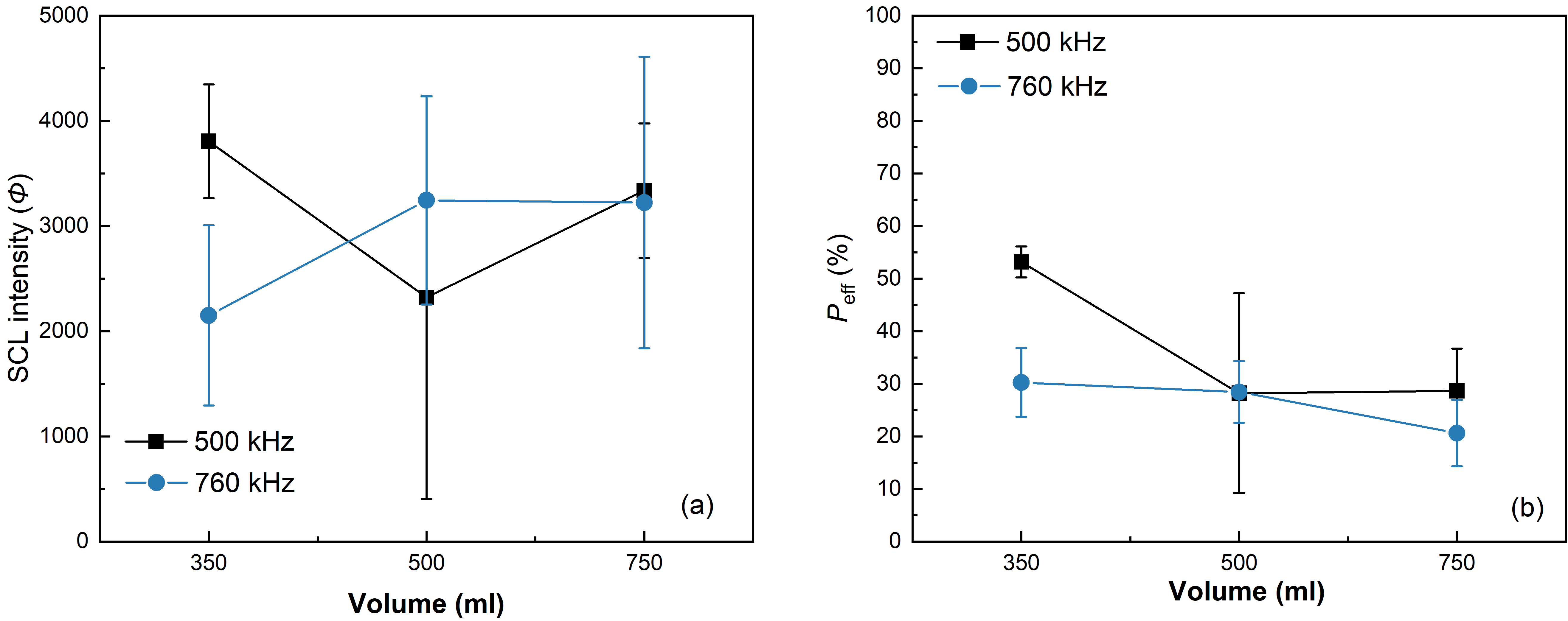


**Fig. S2** Experimental findings with cubic reactor: (a) SCL intensity (*Φ*), and (b) effective SCL proportion (*P*_eff_)

**Appendix C**

**SCL image quality review under typical operating conditions**


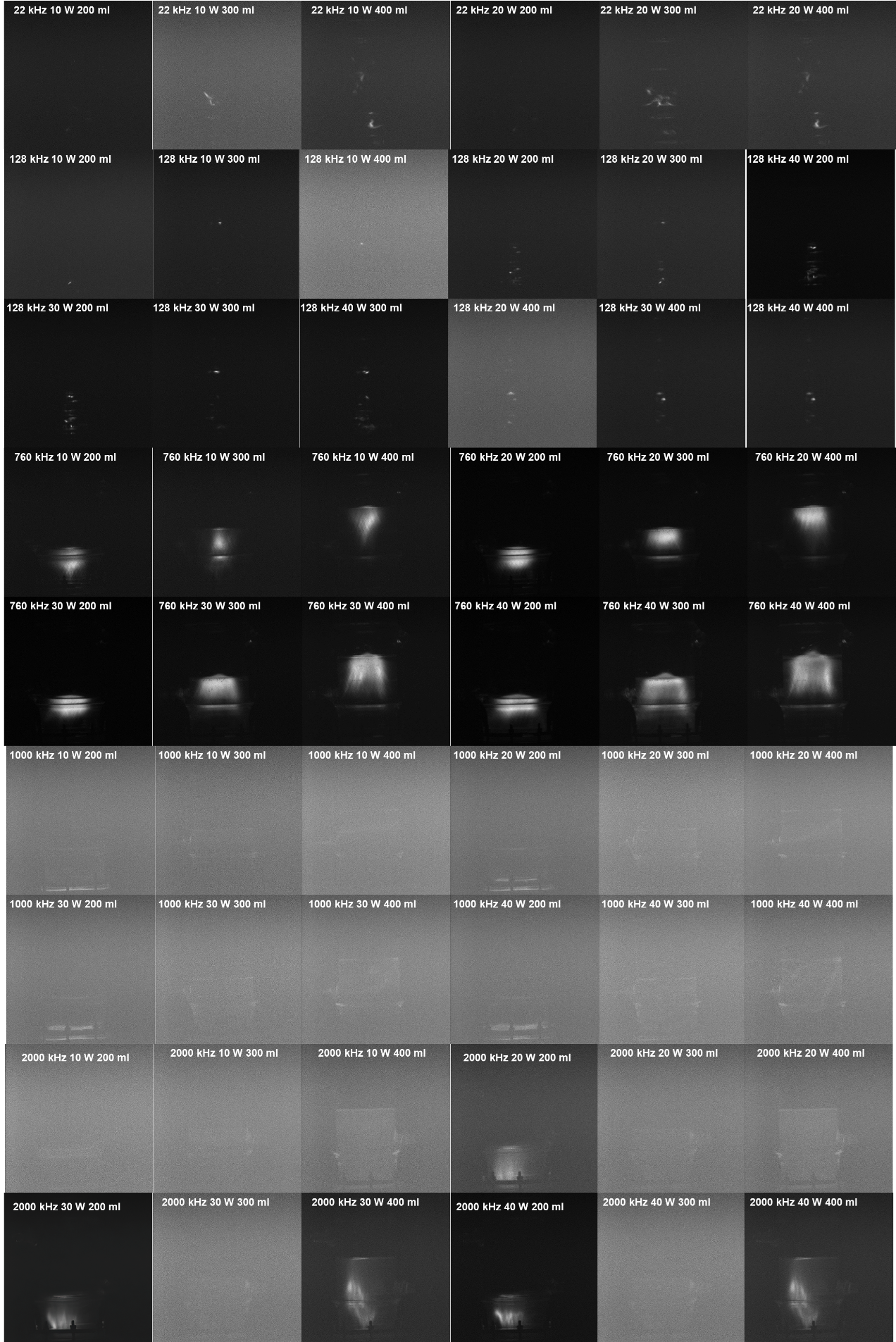


**Fig. S3** SCL emissions captured at various frequencies under all tested powers and volumes (critical frequencies: 22, 128, 1000, and 2000 kHz; control frequency: 760 kHz).

**Appendix D**

**Modelling of SCL intensity (*Φ*)**


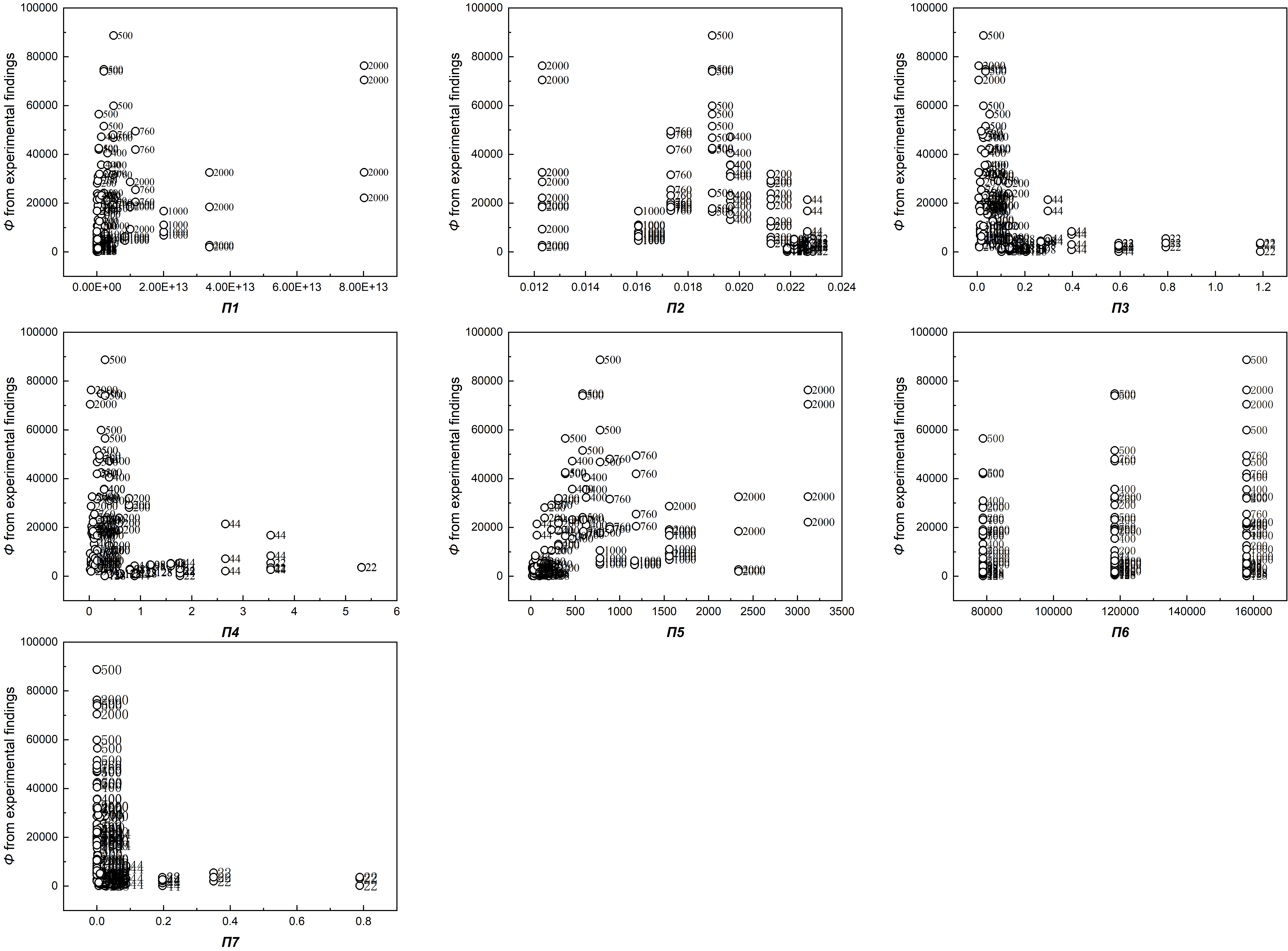


**Fig. S4.** Trend of SCL intensity, *Φ* with each dimensionless number from SCL measurement: (a)*Π1,* (b) *Π2,* (c) *Π3,* (d) *Π4,* (e) *Π5,* (f) *Π6* and (g) *Π7* (the frequency (kHz) is labelled)

*
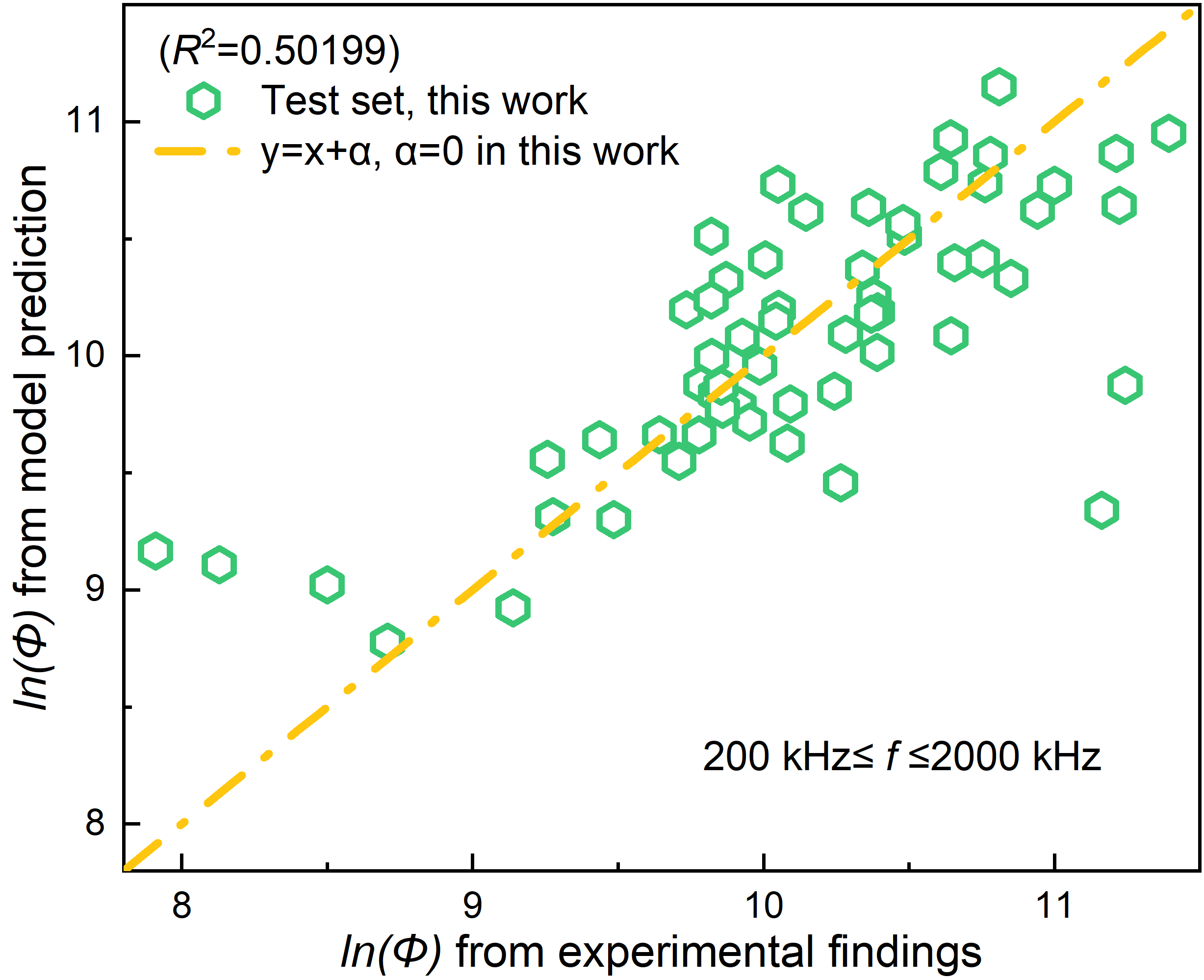
*

**Fig. S5** Comparison of the experimental and predicted results about *Φ* (200 kHz≤ f ≤2000 kHz)

**Table S2.** Results of the MLR analysis about *Φ* at 200 kHz*≤ f ≤*2000 kHz

| **Parameter** | **Correlation coefficient** | **Parameter** | **Correlation coefficient** | **R^2^** |
| --- | --- | --- | --- | --- |
| ln*K* | -19653.14813 | *x4* | 0.77234 | 0.50199 |
| *x1* | 22.93352 | *x5* | 8783.33044 |  |
| *x2* | 6.48873 | *x6* | -24.75484 |  |
| *x3* | -15168.5409 | *x7* | 11997.65802 |  |

**Appendix E**

**Modelling of effective SCL proportion (*P_eff_*)**


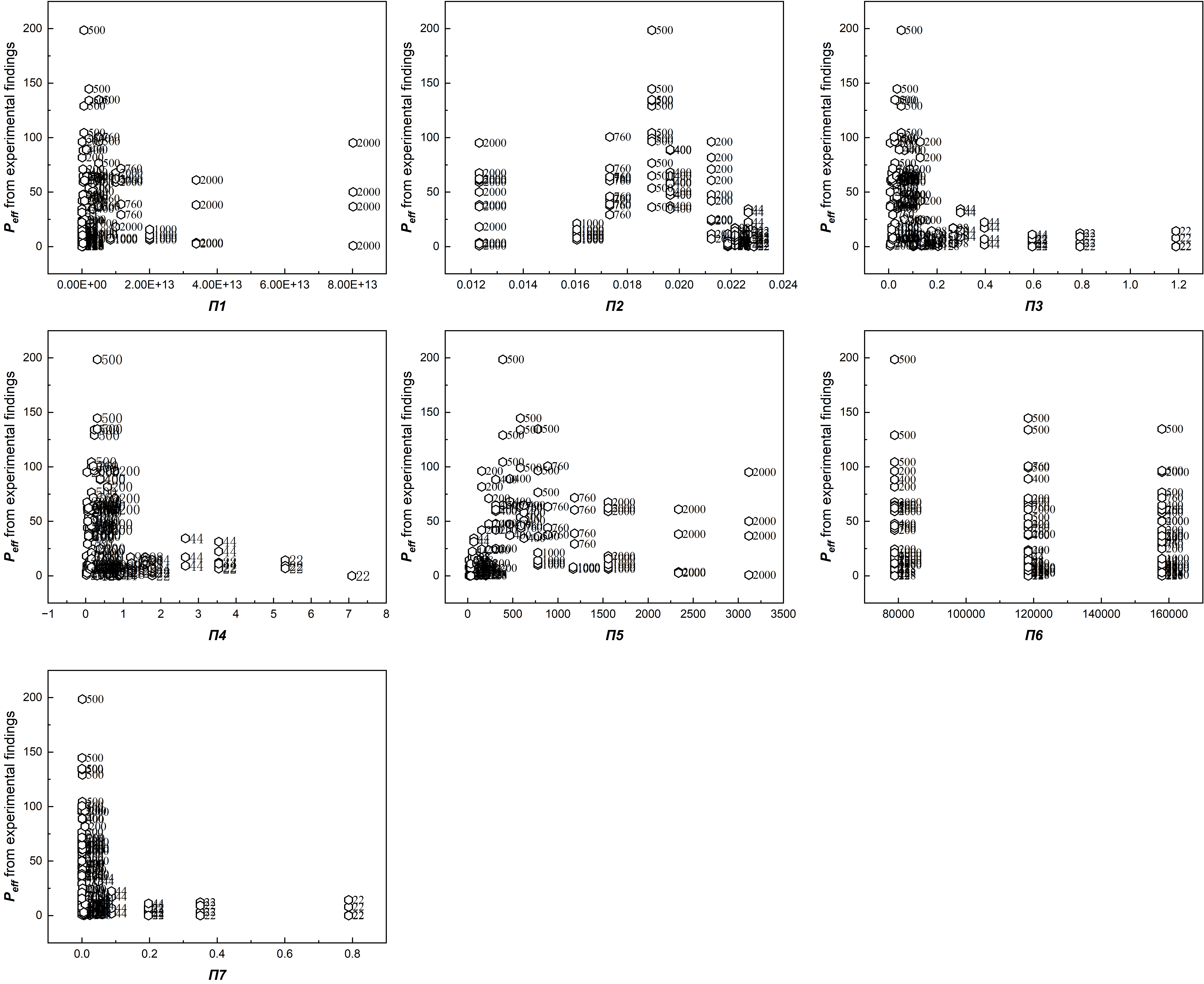


**Fig. S6.** Trend of effective SCL proportion, *P_eff_* with each dimensionless number from SCL measurement: (a)*Π1,* (b) *Π2,* (c) *Π3,* (d) *Π4,* (e) *Π5,* (f) *Π6* and (g) *Π7* (the frequency (kHz) is labelled)
